# Genetic variation in Aquaporin-4 moderates the relationship between sleep and brain Aβ-amyloid burden

**DOI:** 10.1038/s41398-018-0094-x

**Published:** 2018-02-26

**Authors:** Stephanie R. Rainey-Smith, Gavin N. Mazzucchelli, Victor L. Villemagne, Belinda M. Brown, Tenielle Porter, Michael Weinborn, Romola S. Bucks, Lidija Milicic, Hamid R. Sohrabi, Kevin Taddei, David Ames, Paul Maruff, Colin L. Masters, Christopher C. Rowe, Olivier Salvado, Ralph N. Martins, Simon M. Laws, for the AIBL Research Group

**Affiliations:** 1 Centre of Excellence for Alzheimer’s Disease Research and Care, School of Medical and Health Sciences, Edith Cowan University, Joondalup, 6027 WA Australia; 2 Sir James McCusker Alzheimer’s Disease Research Unit, Hollywood Private Hospital, Perth, 6009 WA Australia; 3 Collaborative Genomics Group, Centre of Excellence for Alzheimer’s Disease Research and Care, School of Medical and Health Sciences, Edith Cowan University, Joondalup, 6027 WA Australia; 4 The Florey Institute of Neuroscience and Mental Health, The University of Melbourne, Parkville, 3052 VIC Australia; 5 Department of Nuclear Medicine and Centre for PET, Austin Health, Heidelberg, 3084 VIC Australia; 6 School of Psychology and Exercise Science, Murdoch University, Murdoch, 6150 WA Australia; 7 Co-operative Research Centre for Mental Health, Carlton, VIC Australia; 8 School of Psychological Science, University of Western Australia, Crawley, 6009 WA Australia; 9 Department of Biomedical Sciences, Macquarie University, North Ryde, 2113 NSW Australia; 10 Academic Unit for Psychiatry of Old Age, St. Vincent’s Health, The University of Melbourne, Kew, 3101 VIC Australia; 11 National Ageing Research Institute, Parkville, 3052 VIC Australia; 12 CogState Ltd., Melbourne, 3000 VIC Australia; 13 CSIRO Health and Biosecurity/Australian e-Health Research Centre, Herston, 4029 QLD Australia; 14 School of Biomedical Sciences, Faculty of Health Sciences, Curtin Health Innovation Research Institute, Curtin University, Bentley, 6102 WA Australia

## Abstract

The glymphatic system is postulated to be a mechanism of brain Aβ-amyloid clearance and to be most effective during sleep. Ablation of the astrocytic end-feet expressed water-channel protein, Aquaporin-4, in mice, results in impairment of this clearance mechanism and increased brain Aβ-amyloid deposition, suggesting that Aquaporin-4 plays a pivotal role in glymphatic function. Currently there is a paucity of literature regarding the impact of *AQP4* genetic variation on sleep, brain Aβ-amyloid burden and their relationship to each other in humans. To address this a cross-sectional observational study was undertaken in cognitively normal older adults from the Australian Imaging, Biomarkers and Lifestyle (AIBL) study. Genetic variants in *AQP4* were investigated with respect to self-reported Pittsburgh Sleep Quality Index sleep parameters, positron emission tomography derived brain Aβ-amyloid burden and whether these genetic variants moderated the sleep-Aβ-amyloid burden relationship. One *AQP4* variant, rs72878776, was associated with poorer overall sleep quality, while several SNPs moderated the effect of sleep latency (rs491148, rs9951307, rs7135406, rs3875089, rs151246) and duration (rs72878776, rs491148 and rs2339214) on brain Aβ-amyloid burden. This study suggests that *AQP4* genetic variation moderates the relationship between sleep and brain Aβ-amyloid burden, which adds weight to the proposed glymphatic system being a potential Aβ-amyloid clearance mechanism and suggests that *AQP4* genetic variation may impair this function. Further, *AQP4* genetic variation should be considered when interpreting sleep-Aβ relationships.

## Introduction

Estimates suggest that dysfunctional sleep may be present in up to 45% of Alzheimer’s disease (AD) patients^1^; manifesting commonly as frequent awakenings, increased sleep latency (time to fall asleep) and poor sleep maintenance^2^. However, accumulating evidence also suggests that there is a bi-directional relationship between sleep and AD phenotypes^3–5^; i.e., in addition to the AD phenotype leading to sleep dysfunction, dysfunctional sleep contributes to the AD phenotype.

Aβ-amyloid (Aβ) accumulates gradually in the brain of individuals as they progress towards a diagnosis of AD^6^. This accumulation of Aβ is thought to begin about 20 years before the onset of AD symptomology^7^, and in the sporadic form of the disease, is hypothesized to be driven by poor clearance mechanisms^6^. The mechanisms of Aβ clearance from the human brain are multiple, with some specific factors involved yet to be fully understood^8^. Significantly, however, it has been observed in mice that good quality sleep enhances brain Aβ clearance^9^, while dysfunctional sleep exacerbates Aβ accumulation in humans^10^ and animal models^11^. Further, we have recently shown that in cognitively normal older adults, increased sleep latency is associated with higher brain Aβ burden^12^. It is hypothesized that the brain has a lymphatic-like clearance system that operates parallel to the human lymphatic system through the employment of a network of paravascular clearing mechanisms^13^. This lymphatic-like clearance system has been termed the glymphatic system^14,15^, and is postulated to function almost entirely during sleep^9^.

Evidence from animal models supports a cerebral perivascular and paravascular clearing mechanism that involves the bulk flow of interstitial fluid and the involvement of the water-channel protein, Aquaporin-4 (AQP4)^16^, located primarily in the subpial and perivascular end-feet of astrocytic processes. Further, evidence from *Aqp4* gene knockout mice supports the notion that the functionality of AQP4 is related to the efficacy of Aβ clearance^17^, likely via the glymphatic system^18^. Additionally, a study of autopsied human brains observed that AQP4 immunoreactivity is distributed in a manner similar to neuritic Aβ plaques^19^; suggesting that AQP4 is likely to be linked to Aβ plaque deposition in the brain^20^. Further, a decrease in AQP4 expression or loss of perivascular localization could contribute to reduced Aβ clearance^21^.

Rare in silico-predicted functional variants have been identified in human *AQP4*, which have been shown to impair water permeability *in vitro*^22^. However, no studies to date have investigated the role of *AQP4* genetic variation in AD, sleep and Aβ clearance. To deepen our understanding of the role of AQP4 in AD, we investigated genetic variation across the *AQP4* gene with respect to the relationship with, and between, sleep quality/quantity and brain Aβ burden. We hypothesized that poorer quality sleep would be associated with higher Aβ brain burden and that genetic variants in *AQP4* would moderate this relationship. This hypothesis was derived from the premise that both poor sleep quality decreases Aβ clearance (i.e., results in higher brain Aβ burden), and also that sub-optimal glymphatic clearance during dysfunctional sleep will result in a higher cerebral Aβ burden; *ergo*, *AQP4* genetic variation would have a functional impact on brain Aβ burden.

## Materials and methods

### Study participants

This cross-sectional investigation utilized data collected from Cognitively Normal (CN) older adults of the Australian Imaging, Biomarkers and Lifestyle (AIBL) Study; a prospective longitudinal study of ageing launched in 2006. All volunteers were aged 60 years and above at baseline. Further details regarding the design, enrolment process, neuropsychological assessments, and diagnostic criteria of the AIBL Study have been previously described elsewhere^23^. The AIBL Study is approved by the institutional ethics committees of Austin Health, St Vincent’s Health, Hollywood Private Hospital, and Edith Cowan University (ECU), and informed written consent was given by all volunteers.

### Sleep measures

Subjective sleep quality and disturbances were assessed via the Pittsburgh Sleep Quality Index (PSQI)^24^ in 462 CN older adults at the 72-month time point of the study. Several parameters are subsequently derived from this 19-item self-report measure, including sleep quality, latency (in min), duration (reported in hours), efficiency, sleep disturbance, medication use and daytime dysfunction. A further global score of sleep quality, PSQI Total, is also derived, with a score > 5 indicating poor sleep. For the present study, analyses focused on parameters related to night-time function and thus, the factors studied herein were limited initially to overall sleep quality (PSQI Total), then subsequently to the sub-scales of sleep latency, sleep duration, sleep efficiency, and sleep disturbances.

### Brain imaging

Of these 462 participants, 222 also underwent Αβ imaging. Αβ imaging was performed via positron emission tomography (PET) using one of the following radiolabeled Αβ tracers; ^11^C-Pittsburgh Compound B (PiB), ^18^F-florbetapir or ^18^F-flutemetamol, as previously described^25–27^. Images were analyzed using CapAIBL, a web-based freely available magnetic resonance (MR)-less methodology, to generate PET standardized uptake value (SUV) ratios (SUVR) for all tracers^28^. Briefly, SUVs were summed and normalized to either the cerebellar cortex SUV (PiB), whole cerebellum SUV (florbetapir), or pons SUV (flutemetamol), to yield the target-region to reference-region SUVR. To allow for the analysis of these different tracers as a single continuous variable, a linear regression transformation was applied to generate PiB-like SUVR units termed the ‘Before the Centiloid Kernel Transformation’ (BeCKeT) scale^29^. PiB SUVR and florbetapir/flutemetamol BeCKeT were utilized in this cross-sectional study.

### Genetic data

Genetic data were derived from a genome-wide single-nucleotide polymorphism (SNP) array conducted on the Illumina OmniExpressHumanExome + BeadChip with subsequent imputation using impute2 ver2.3, with the 1000 genome reference panel (2015 release). SNP data from the *AQP4* genomic region (GRCh37 Chr18:24,432,000–24,446,000) were extracted and subjected to quality control in GoldenHelix SNP and Variation Suite (SVS version 8.7.1), which included removal of SNPs with call rate < 95%, Minor allele frequency < 5% and departure from Hardy–Weinberg Equilibrium (*p* < 0.05), leaving 32 SNPs (Supplementary Table 1). After Linkage Disequilibrium (LD) pruning (*r*^2^ cutoff of 0.8, window size 10, increment 5), 13 *AQP4* SNPs were selected to provide full coverage of the gene (Supplementary Table 1, Supplementary Figure 1). Apolipoprotein E (*APOE*) genotype, specifically the presence of the ε4 allele, the major genetic risk factor for AD, was determined using TaqMan genotyping assays (Life Technologies, USA) for rs7412 (Assay ID: C____904973_10) and rs429358 (Assay ID: C___3084793_20) on a QuantStudio 12 K Flex real-time PCR system (Applied Biosystems, USA).

### Statistical analysis

Statistical analyses were carried out in either Golden Helix (Inc.) SVS (version 8.7.1) for linear regression analyses, using additive (homozygote for the minor allele (MM) vs. heterozygote for the minor allele (Mm) vs. homozygote for the major allele (mm)), recessive (homozygote for the minor allele (MM) vs. heterozygote/homozygote for the major allele (Mm/mm)) and dominant (heterozygote/homozygote for the minor allele (Mm or MM) vs. homozygote for the major allele (mm)) genetic models, or IBM SPSS Statistics, Version 24.0 (IBM Corp., Armonk, NY) for moderation analyses, using recessive and dominant models only. Nominal significance (uncorrected) was reported at *p* < 0.05. However, final levels of significance were ascertained after correction for the False Discovery Rate (FDR) with significance threshold set at *q* < 0.05^30^. Linear regression analysis, with respect to Aβ burden, included the covariates of age, sex, and *APOE* genotype (presence/absence of the ε4 allele). For the PSQI sleep parameters, body mass index (BMI), depressive symptomology (Geriatric depression Scale; GDS) and a medical history of cardiovascular disease (CVD) were also included as covariates. The relationship between *AQP4* SNPs and PSQI sleep parameters was undertaken using a two-stage approach. First, the association with overall sleep quality (PSQI total) was assessed followed by analysis of association with the PSQI sub-scales (sleep latency, duration, efficiency and disturbances).

Moderation analysis in SPSS utilized a custom dialog: PROCESS (release 2.16.3)^31^ with 5000 bias-corrected and accelerated bootstrap samples, with 95% confidence intervals. *AQP4* SNPs were included as the moderator variable (*W*), brain Αβ burden as the outcome variable (*Y*), with each of the five selected PSQI sleep parameters entered individually as the independent variable (*X*). Moderation analyses covaried for age, BMI, medical history of CVD, GDS and *APOE* ε4 allele carriage as previously reported^12^. Post hoc simple slopes analysis was used to visualize the moderation of the effect of *X* on *Y* by the moderating variable, *W*^32^

## Results

Demographic characteristics for the study cohort are presented in Table 1. Neuroimaging data were only available in 222 CN older adults at the same assessment time point at which the PSQI was administered. However, there were no significant differences in terms of the distributions or means of the demographic variables between the PSQI only group (*n* = 462) and the PSQI plus imaging subset (*n* *=* 222).

**Table 1:** Cohort demographics

	PSQI Only	PSQI and Aβ
*n*	462	222
Age, years	75.0 ± 6.0	75.2 ± 6.1
Sex, % Female	58.1	57.2
*APOE*, % ε4 carriers	22.7	23
Aβ (SUVR/BeCKeT^a^)	1.38 ± 0.38^b^	1.38 ± 0.38
Time between PSQI and PET scan (days)		173.7 ± 132.3
MMSE	28.9 ± 1.3	28.9 ± 1.4
BMI (kg/m^2^)	26.5 ± 4.3	26.4 ± 4.2
GDS	1.4 ± 1.7	1.3 ± 1.6
% Good sleepers^c^ (n)	50.9 (235)	55.9 (124)
PSQI Total	6.2 ± 1.2	5.6 ± 3.2
Sleep latency (minutes)	19.9 ± 19.4	17.0 ± 16.6
Sleep duration (hours)	6.8 ± 1.2	7.0 ± 1.2

^a11^C-Pittsburgh compound B PET (PiB-PET) like standardized uptake value ratio (SUVR) generated using the Before the Centiloid Kernel Transformation (BeCKeT) scale

^b^ *n* = 222

^c^Good sleeper, defined by PSQI Total score ≤ 5

All values represented as mean ± s.d., unless otherwise indicated. *Aβ* Aβ-amyloid; *APOE* apolipoprotein E ε4 allele carriage; *BMI* body mass index; *GDS* Geriatric Depression Scale; *MMSE* Mini Mental State Examination; *PET* Positron Emission Tomography; *PSQI* Pittsburgh Sleep Quality Index

### *AQP4* genetic variation and PSQI sleep parameters or brain Aβ burden

Linear regression analysis was performed to determine whether *AQP4* SNPs were associated firstly with overall sleep quality (PSQI total) and subsequently with the PSQI sub-scales (sleep latency, sleep duration, sleep efficiency, and sleep disturbances), using both a base statistical model (no covariates) and an adjusted statistical model, covarying for age, BMI, medical history of CVD, GDS and *APOE* ε4 allele carriage. Nominal significance (Table 2) was observed with respect to PSQI Total (rs71353406, rs72878776, and rs3875089) and subsequently, in the sub-scale analyses, with sleep disturbances (rs68006382). Of these associations, only that of rs72878776 with PSQI Total (base model, *β* = 4.74 (s.e.: 1.37), *p* = 0.0006; adjusted model, *β* = 4.15 (s.e.: 1.34), *p* = 0.002*)* remained significant after FDR correction (base model, *q* = 0.008; adjusted model, *q* = 0.028). No further associations were observed for remaining genetic variants and PSQI sleep parameters (Supplementary Table 2).

**Table 2:** Association of *AQP4* SNPs with Pittsburgh Sleep Quality Index sleep parameters

PSQI sleep parameter	SNP Ref	Additive^a^		Dominant^a^		Recessive^a^	
		*Base* ^b^	*Adj* ^b^	*Base* ^b^	*Adj* ^b^	*Base* ^b^	*Adj* ^b^
PSQI Total	rs71353406	0.130	0.100	**0.042**	**0.045**	0.856	0.871
	rs72878776	0.593	0.836	0.647	0.466	**0.001** ^c^	**0.002** ^c^
	rs3875089	0.494	0.442	0.940	0.931	**0.012**	**0.021**
Sleep disturbances	rs68006382	0.097	0.146	**0.034**	0.077	0.062	0.902

^a^Genetic models: Additive (homozygote for the minor allele (MM) vs. heterozygote for the minor allele (Mm) vs. homozygote for the major allele (mm)); Recessive (homozygote for the minor allele (MM) vs. heterozygote/homozygote for the major allele (Mm/mm)); Dominant (heterozygote/homozygote for the minor allele (Mm or MM) vs. homozygote for the major allele (mm))

^b^Statistical models: *Base *base statistical model including no covariates, *Adj *Adjusted statistical model (covaries for: age, sex, body mass index (BMI), geriatric depression scale (GDS) and a medical history of CVD). Values that reached nominal significance (*p* < 0.05, uncorrected) are bolded

^c^values significant after False Discovery Rate correction (*q* < 0.05)

Summary of Aquaporin-4 (*AQP4*) SNPs demonstrating significant associations with sleep parameters. *PSQI* Pittsburgh Sleep Quality Index Sleep Parameters: PSQI Total, sleep disturbances. SNP Ref, reference single-nucleotide polymorphism marker (rs); *AQP4* Aquaporin-4

Linear regression analyses were also performed to determine whether *AQP4* SNPs were associated with brain Aβ burden, again using both a base statistical model (no covariates) and an adjusted statistical model, covarying for age, sex and *APOE* ε4 allele carriage. No significant associations were observed between *AQP4* genetic variants and brain Aβ burden either independently or when the covariates of age, sex, and *APOE* ε4 were included in the adjusted models (Supplementary Table 3).

### *AQP4* moderation of PSQI sleep parameter—brain Aβ burden relationship

Linear regression analysis (Supplementary Table 4), excluding *AQP4* SNPs, revealed that sleep latency (minutes) was associated with Aβ burden (*β* = 0.004, *t*(215) = 2.66; 95% CI, 0.001–0.007; *p* = 0.008), consistent with a previous report in a subset of this same sample^12^. No other PSQI sleep parameter was observed to be associated with brain Aβ burden in our analyses.

**Fig. 1:**
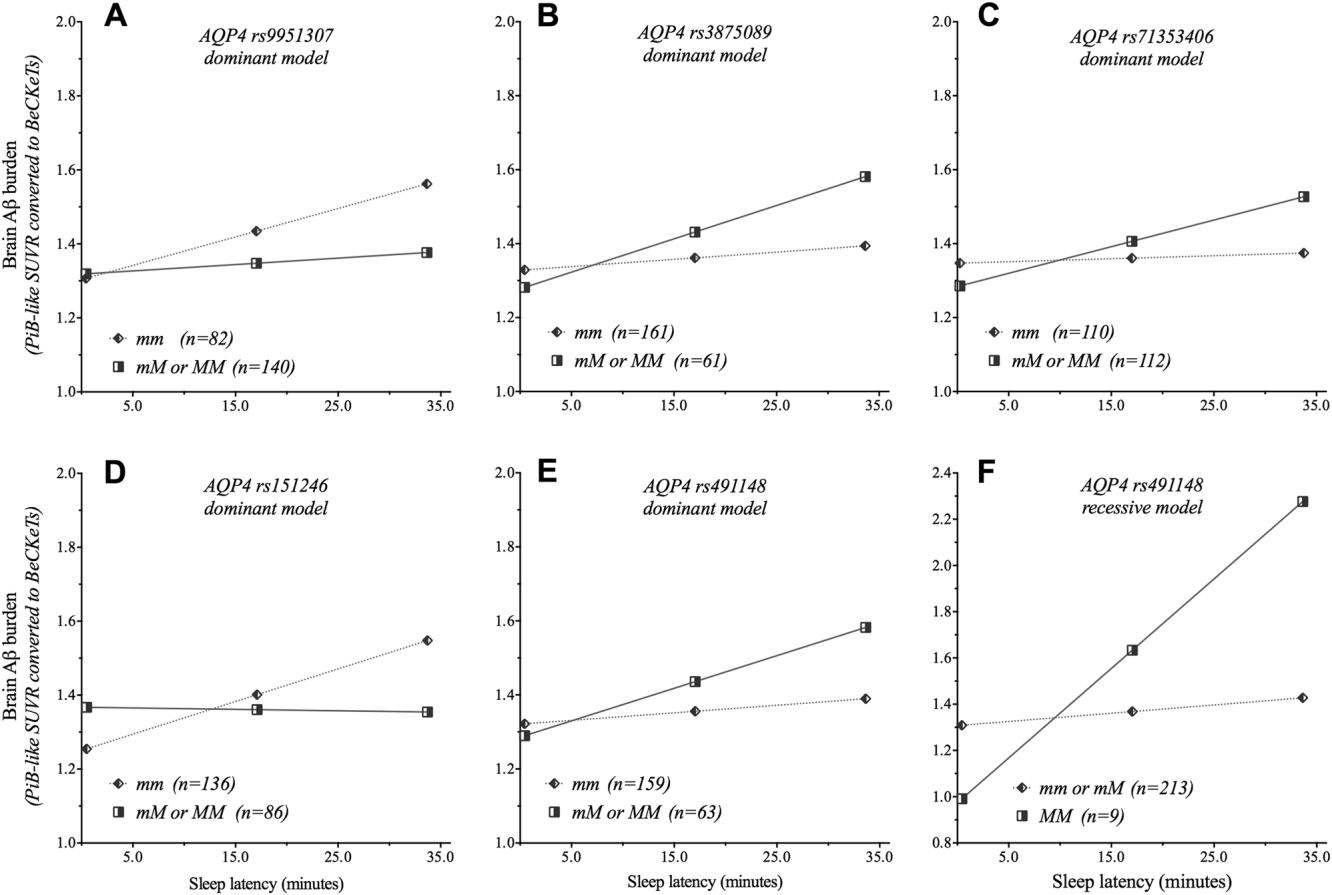
Conditional effects of *AQP4* SNPs on the relationship between sleep latency and brain Aβ burden. Moderating effects of the Aquaporin-4 (*AQP4*) single-nucleotide polymorphisms (SNPs) (**A**) rs9951307 (dominant model), (**B**) rs3875089 (dominant model), (**C**) rs7135406 (dominant model), (**D**) rs151246 (dominant model) and rs491148, for both the (**E**) dominant and (**F**) recessive genetic models, on the relationship between sleep latency (min) and brain Aβ burden. *M* Minor allele, *m* major allele. Dominant genetic model: homozygote for the major allele (mm) compared to heterozygote/homozygote for the minor allele (mM or MM). Recessive genetic model: homozygote/heterozygote for the major allele (mm or mM) compared to homozygote for the minor allele (MM). Brain Aβ burden is presented as ^11^C-Pittsburgh compound B (PiB) positron emission tomography (PET)-like standardized uptake value ratio (SUVR) and as the Before the Centiloid Kernel Transformation (BeCKeT) scale for florbetapir and flutemetamol studies

To determine whether *AQP4* SNPs moderated the relationship between the 5 PSQI sleep parameters and brain Aβ burden, multivariate linear regression analyses were performed within the moderation model. Nine of these statistical models revealed significant moderation effects for *AQP4* SNPs on the PSQI sleep parameters of sleep latency and sleep duration (Table 3). The relationship between PSQI-determined sleep latency and brain Aβ burden was observed to be significantly moderated by a total of five *AQP4* SNPs. The interaction of the *AQP4* SNPs rs491148 and sleep latency was statistically significant for both dominant (*R*^2^-change* (*Δ*R*^*2*^) = 0.017; *p* = 0.036) and recessive (Δ*R*^*2*^ = 0.020; *p* = 0.022) genetic models. While rs9951307 (Δ*R*^2^ = 0.015; *p* = 0.048), rs71353406 (Δ*R*^2^ = 0.019; *p* = 0.030), rs3875089 (Δ*R*^2^ = 0.019; *p* = 0.028) and rs151246 (Δ*R*^2^, 0.039; *p* = 0.002) were significant in the dominant genetic model only. Post hoc simple slopes analyses (Fig. 1) revealed that for rs3875089, rs71353406, and rs491148, carriage of at least one copy of the minor allele was associated with higher brain Aβ burden as sleep latency increased, while for rs9951307 and rs151246 this relationship was observed for homozygosity of the major allele. All results from moderation analyses are presented in Supplementary Tables 5–9.

**Table 3:** Moderation analysis for *AQP4* SNPs on sleep latency and sleep duration

	Dominant^a^						Recessive^a^					
	*β*	s.e.	Sig.	*R* ^2^	Sig.	Δ*R*^2^	*β*	s.e.	Sig.	*R* ^2^	Sig.	Δ*R*^2^
*LATENCY*												
*Model summary: rs151246*				0.201	<0.001					0.165	<0.001	
Age	0.009	0.004	0.023				0.011	0.004	0.012			
BMI	0.004	0.006	0.525				0.005	0.006	0.448			
CVD risk	−0.015	0.038	0.699				−0.014	0.039	0.731			
GDS	−0.007	0.015	0.654				−0.011	0.015	0.469			
*APOE* ε4	0.303	0.056	<0.001				0.310	0.057	<0.001			
rs151246	0.117	0.070	0.096				0.064	0.175	0.716			
Latency	0.009	0.002	<0.001				0.004	0.002	0.006			
INT	−0.009	0.003	0.002			**0.039**	−0.008	0.007	0.294			0.004
*Model summary: rs9951307*				0.186	<0.001					0.166	<0.001	
Age	0.010	0.004	0.019				0.010	0.004	0.013			
BMI	0.003	0.006	0.607				0.004	0.006	0.752			
CVD risk	−0.013	0.039	0.741				−0.012	0.039	0.752			
GDS	−0.007	0.015	0.658				−0.010	0.015	0.485			
*APOE* ε4	0.312	0.056	<0.001				0.309	0.057	<0.001			
rs9951307	0.015	0.070	0.831				0.025	0.123	0.837			
Latency	0.008	0.002	0.001				0.004	0.002	0.006			
INT	−0.006	0.003	0.048			**0.015**	−0.005	0.006	0.347			0.004
*Model summary: rs71353406*				0.180	<0.001					0.163	<0.001	
Age	0.010	0.004	0.023				0.010	0.004	0.018			
BMI	0.004	0.006	0.556				0.005	0.006	0.401			
CVD risk	−0.008	0.039	0.833				−0.012	0.040	0.769			
GDS	−0.006	0.015	0.692				−0.009	0.015	0.552			
*APOE* ε4	0.298	0.058	<0.001				0.307	0.058	<0.001			
rs71353406	−0.063	0.069	0.362				0.050	0.158	0.754			
Latency	0.001	0.002	0.688				0.004	0.002	0.022			
INT	0.006	0.003	0.030			**0.019**	0.003	0.006	0.675			0.001
*Model summary: rs3875089*				0.184	<0.001					0.165	<0.001	
Age	0.010	0.004	0.019				0.011	0.004	0.010			
BMI	0.005	0.006	0.400				0.004	0.006	0.458			
CVD risk	−0.016	0.039	0.683				−0.017	0.040	0.660			
GDS	−0.010	0.015	0.501				−0.012	0.015	0.426			
*APOE* ε4	0.310	0.057	<0.001				0.313	0.058	<0.001			
rs3875089	−0.050	0.074	0.497				−0.005	0.416	0.990			
Latency	0.002	0.002	0.248				0.004	0.002	0.008			
INT	0.007	0.003	0.028			**0.019**	0.010	0.027	0.706			0.001
*Model summary: rs491148*				0.185	<0.001					0.193	<0.001	
Age	0.010	0.004	0.016				0.012	0.004	0.005			
BMI	0.005	0.006	0.360				0.005	0.006	0.393			
CVD risk	−0.018	0.039	0.650				−0.017	0.039	0.657			
GDS	−0.011	0.015	0.450				−0.011	0.015	0.459			
*APOE* ε4	0.316	0.057	<0.001				0.320	0.057	<0.001			
rs491148	−0.035	0.075	0.639				−0.333	0.271	0.220			
Latency	0.002	0.002	0.639				0.004	0.001	0.014			
INT	0.007	0.003	0.036			**0.017**	0.035	0.015	0.022			**0.020**
*DURATION*												
*Model summary: rs72878776*				0.149	<0.001					0.126	<0.001	
Age	0.012	0.004	0.005				0.011	0.004	0.010			
BMI	0.005	0.006	0.370				0.004	0.006	0.520			
CVD risk	−0.023	0.040	0.565				−0.009	0.040	0.816			
GDS	−0.007	0.015	0.662				−0.003	0.015	0.838			
*APOE* ε4	0.289	0.058	<0.001				0.283	0.059	<0.001			
rs12968026	0.807	0.352	0.023				0.065	0.715	0.928			
Duration	0.026	0.023	0.251				0.005	0.021	0.817			
INT	−0.104	0.049	0.034			**0.019**	−0.010	0.105	0.923			<0.001
*Model summary: rs2339214*				0.132	<0.001					0.174	<0.001	
Age	0.010	0.004	0.018				0.011	0.004	0.009			
BMI	0.005	0.006	0.403				0.004	0.006	0.507			
CVD risk	−0.011	0.041	0.796				−0.009	0.040	0.819			
GDS	−0.005	0.016	0.774				−0.008	0.015	0.595			
*APOE* ε4	0.302	0.059	<0.001				0.307	0.058	<0.001			
rs2339214	0.056	0.324	0.864				−0.993	0.329	0.003			
Duration	0.014	0.038	0.714				−0.031	0.024	0.197			
INT	−0.009	0.045	0.850			<0.001	0.149	0.047	0.002			**0.041**
*Model Summary: rs491148*					0.156	<0.001					0.146	
Age	0.011	0.004	0.007				0.012	0.004	0.005			
BMI	0.005	0.006	0.377				0.004	0.006	0.736			
CVD risk	−0.023	0.040	0.565				−0.016	0.040	0.684			
GDS	−0.012	0.015	0.419				−0.008	0.015	0.574			
*APOE* ε4	0.317	0.058	<0.001				0.316	0.059	<0.001			
rs491148	0.707	0.320	0.028				−0.135	0.662	0.839			
Duration	0.030	0.024	0.202				0.005	0.021	0.819			
INT	−0.090	0.045	0.045			**0.016**	0.053	0.097	0.584			0.001

^a^Genetic models: Dominant (heterozygote/homozygote for the minor allele (Mm or MM) vs. homozygote for the major allele (mm)), Recessive (homozygote for the minor allele (MM) vs. heterozygote/homozygote for the major allele (Mm/mm)); *β* coefficient of predictors; *Sig* *p*-value; *R*^2^ coefficient of multiple determination; Δ*R*^2^ multiple correlation coefficient (*R*) squared change; *APOE* Apolipoprotein E ε4 allele carriage (presence/absence); *BMI* body mass index; *CVD risk* cardiovascular disease risk; *GDS* Geriatric Depression Scale; *INT* Interaction (Sleep Latency/Duration × model summary SNP). Models where the interaction term (INT) resulted in a statistically significant *R*^2^-change (*p* < 0.05) are indicated (bolded)

Model summary statistics for significant Aquaporin-4 (*AQP4*) reference single-nucleotide polymorphism (SNP) markers (rs)

**Fig. 2:**
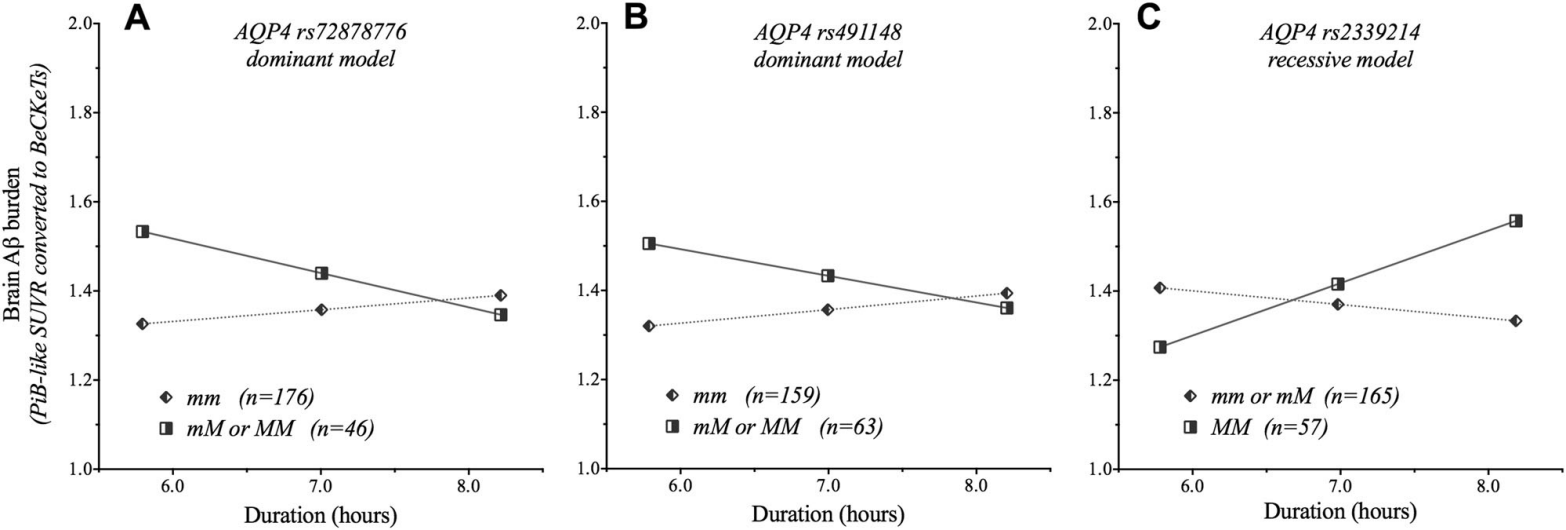
Conditional effects of *AQP4* SNPs on the relationship between sleep duration and brain Aβ burden. Moderating effects of Aquaporin-4 (*AQP4*) single-nucleotide polymorphisms (SNPs) (**A**) rs72878776 (dominant model), (**B**) rs491148 (dominant model), and (**C**) rs2339214 (recessive model) on the relationship between sleep duration (hours) and brain Aβ burden. M, Minor allele; m, major allele. Dominant genetic model: homozygote for the major allele (mm)) compared to heterozygote/homozygote for the minor allele (mM or MM). Recessive genetic model: homozygote/heterozygote for the major allele (mm or mM) compared to homozygote for the minor allele (MM). Brain Aβ burden is presented as ^11^C-Pittsburgh compound B (PiB) positron emission tomography (PET) like standardized uptake value ratio (SUVR) and as the Before the Centiloid Kernel Transformation (BeCKeT) scale for florbetapir and flutemetamol studies

Three *AQP4* SNPs interacted with PSQI-determined sleep duration (in hours) to significantly impact brain Aβ burden, namely rs72878776, rs2339214 and rs491148 (Table 3). For rs72878776 (Δ*R*^*2*^ = 0.019; *p* = 0.034) and rs491148 (Δ*R*^*2*^ = 0.016; *p* = 0.045) the association was observed in the dominant genetic model, while for rs2339214 (Δ*R*^*2*^ = 0.041; *p* = 0.002), the association was observed in the recessive model. Post hoc simple slopes analyses (Fig. 2) revealed that for rs72878776 and rs491148, carriage of at least one copy of the minor allele resulted in higher brain Aβ burden with a shorter duration of sleep. However, the opposite was observed for rs2339214, where homozygosity of the minor allele resulted in higher brain Aβ burden with a longer duration of sleep.

## Discussion

The primary aim of this study was to determine whether genetic variation within *AQP4* moderated the relationship between PSQI-derived self-reported sleep quality and brain Aβ burden as assessed by PET in cognitively normal older adults of the AIBL Study. This study is the first to report genetic variation in *AQP4* to be both associated with altered, self-reported, ‘overall’ sleep quality (PSQI Total score), and to moderate the relationship between the sleep parameters of latency (time taken to fall asleep), duration (length of sleep), and brain Aβ burden.

The association of sleep latency with increased brain Aβ burden confirms results previously reported in a subset (*n* = 184) of AIBL participants included in the current study (*n* = 222)^12^. In this prior study, no moderation of the sleep latency-Aβ relationship by *APOE* genotype was observed. However, we report in the current study that moderation of this relationship occurs due to variants in a gene encoding a key component of the postulated glymphatic system: Specifically, *AQP4*, which encodes for AQP4, an astrocytic end-feet expressed water-channel protein postulated to be involved in glymphatic-mediated clearance of Aβ^18^.

Our data suggests that genetic variation in *AQP4*, specifically rs72878776, is associated with altered, self-reported, ‘overall’ sleep quality (PSQI Total score), with individuals homozygous for the *AQP4* rs72878776-A allele reporting worse overall sleep compared to those with a different genotype. This SNP is in the 5-prime untranslated region (5′UTR) of the *AQP4* gene and may be of functional relevance through potentially influencing gene transcription, via modification (creation or deletion) of transcription factor binding sites. This is supported by evidence compiled in the RegulomeDB^33^. Specifically, the potential binding of transcriptional regulators such as REST, TRIM28, CTBP2, and ZNF263 are predicted to be affected by this variant. Analysis of the LD structure of the *AQP4* gene for linkage of rs72878776, with other variants with potential functional implications, revealed it to tag rs35248760, a synonymous SNP in exon 1. While rs35248760 does not appear itself to be a SNP that impacts functionality of the protein it encodes, it cannot be ruled out that rs72878776 may also be in linkage with rare non-synonymous variants in exon 1.

Five *AQP4* SNPs (rs9951307, rs7135406, rs3875089, rs151246, and rs491148) in the dominant models, had significant interactions with self-reported sleep latency and the resultant effect on brain Aβ burden. The impact of rs491148 was observably stronger in homozygotes, suggesting a potential gene-dosage effect for the minor allele (rs491148-G). Specifically, carriage of at least one copy of the rs491148-G allele was associated with a PiB-like SUVR approaching 1.6 when time to fall asleep reached 35 min, while homozygosity of the G-allele, albeit in only 9 individuals, was associated with Aβ burdens approaching 2.3 SUVR/BeCKeT at 35 min latency—a level of brain Aβ usually associated with a clinical diagnosis of mild AD^7^. Of note, three of these *AQP4* variants; rs9951307 (D′ 0.99, *r*^2^ 0.07), rs3875089 (D′ 1.00, *r*^2^ 0.64), and rs491148 (D′ 0.94, *r*^2^ 0.49), are in strong LD, but have reduced correlation, with the aforementioned 5′UTR rs72878776. These *AQP4* SNP-sleep latency findings support previous studies which have reported an association of sleep latency with brain Aβ^10,12^. The current study adds evidence that this relationship is likely moderated by genetic variation in the gene encoding the Aquaporin-4 water-channel protein, which is proposed to be involved in glymphatic-mediated Aβ clearance during sleep^34^. Accordingly, those *AQP4* SNPs that impact the relationship between sleep latency and Aβ may predispose those individuals to suboptimal sleep parameters due to higher Aβ burden. Alternatively, as a bi-directional relationship between sleep and Aβ has also been postulated^3,5^, it is conceivable that suboptimal sleep contributes to higher brain Aβ burden, particularly in those potentially genetically predisposed to poorer functioning of Aβ clearance mechanisms. However, it is also conceivable that the association between *AQP4* variants and sleep quality observed in this study may potentially be attributed to mechanisms unrelated to Aβ dynamics. For example, any impact of genetic variation on expression of AQP4 may, through disrupted water molecule conduct, affect intracellular ionic homeostasis, resulting in impaired cellular function or even death. Since AQP4 is enriched in the glial cells of periventricular regions in the hypothalamus, where hypocretin (orexin)-containing neurons are primarily located, it is conceivable that impaired glial function in these regions may result in secondary neuronal damage leading to impaired sleep regulation through subtle hypocretin deficiency. This is observed to a larger extent in cases of narcolepsy where the presence of anti-AQP4 antibodies is observed^35^.

We also identified three *AQP4* SNPs that interacted with sleep duration to have a moderating effect on levels of Aβ in the brain. Two of these, rs72878776 and rs491148, were also associated with overall sleep quality, and moderation of the relationship between sleep latency and Aβ burden in this study, respectively. With respect to sleep duration, carriage of the minor allele for both rs72878776 and rs491148, was associated with higher Aβ burden with a shorter duration of sleep. However, the opposite was observed for the final variant, rs2339214, Specifically, longer sleep duration (rather than shorter duration) was associated with higher brain Aβ (PiB-like SUVR ~ 1.6, > 8 h sleep duration) in individuals homozygote for the minor allele, rs2339214-A. To our knowledge there is no previous report in the literature of a bimodal relationship between sleep duration and brain Aβ burden. However, there is evidence in the literature that such a bimodal relationship exists between sleep duration and cognition. Specifically, both short and long sleep duration are purported to contribute to poorer cognitive function and increased risk of cognitive impairment and AD compared to intermediate sleep duration^36–38^.

In a recent study by Burfeind and colleagues, two of the *AQP4* SNPs described in the current study, rs9951307 and rs3875089, were reported to be associated with altered trajectories of cognitive decline. We have previously reported in the AIBL study that Aβ is associated with cognitive decline^39–43^ and, as discussed above, suboptimal sleep has also been associated with poorer cognitive function^36–38^. As such, it is plausible that the association of *AQP4* genetic variation with cognitive decline described by Burfeind et al., may be mediated through the impact of *AQP4* on brain Aβ. Interestingly, the association with cognitive decline reported by the authors was limited to those with an established clinical diagnosis of AD, in whom a high Aβ burden would be expected, and was not observed in the ‘Pre-AD’ group. Further, while post mortem evaluation of AD pathology was undertaken, global brain Aβ burden was not evaluated pre-mortem; additional investigation is therefore required to fully elucidate the *AQP4*-cognition relationship, particularly during the preclinical stages of AD and with respect to global brain Aβ burden.

The functional implication of the genetic variants reported herein remain poorly understood and it is clear that further study is required to understand the mechanism that underpins these associations. While there is *in silico* evidence to suggest that some variants may impact the binding of transcription factors, there are other putative mechanisms that may play a role in the relationship between *AQP4* genetic variation and Aβ clearance. For example, several associated SNPs are physically, or in LD with other SNPs, located in the putative promoter region of the AQP4-M23 isoform (M23), the smaller of two isoforms of AQP4^44^, the other being AQP4-M1 (M1). It has been reported that an increased ratio of M23:M1 isoforms occurs in AD and is associated with altered perivascular localization of AQP4^21^. With this loss of perivascular localization, a concomitant worsening of Aβ plaque burden was observed^21^ suggesting that genetic variation that alters isoform relative expression may in turn impact Aβ clearance. Further to this, microRNA mediated regulation of *AQP4* expression, particularly of the M1 isoform, has been reported^45^. However, of the SNPs reported to moderate sleep-Aβ relationships in this study, none were located within the putative microRNA binding sites identified previously^45^, or within the putative M1 promoter region itself. More recently, De Bellis et al.^46^ have demonstrated that in addition to M1 and M23 isoforms, AQP4 may be subject to translational readthrough to generate functionally significant C-terminal extended isoforms, termed AQP4ex. However, of all the associated variants reported in this study, only rs9951307 is located at the C-terminal end of *AQP4*, and is ~15 kb downstream of the *AQP4* UGA canonical stop-codon. Further, this variant does not tag any genetic variants in the vicinity of the stop-codon, although linkage with one or multiple rare-variants in this region cannot be ruled out. Additional study is required to determine the impact of any of these variants on AQP4-M1, AQP4-M23, or AQP4ex isoform expression.

While the findings of this study are novel and suggest that genetic variation of *AQP4* moderates the relationship between sleep parameters and brain Aβ burden, there are some limitations that need to be considered. First, this study was observational and utilized a cross-sectional retrospective design; consequently, no conclusions regarding temporal or causal relationships can be drawn. Second, a subjective sleep measure was utilized which relies on the accuracy and fidelity of the respondents. Utilization of an objective measure of sleep such as actigraphy or polysomnography would circumvent the limitation of self-report. Moreover, use of polysomnography, the ‘gold standard’ in differentiating sleep from wake, and in identifying sleep stages, would provide detail regarding the association of sleep architecture with brain Aβ. However, the PSQI has demonstrated internal consistent reliability and construct validity^47^ and is justified in this study due to its cost effectiveness and ease of administration to a large cohort. Third, the brain imaging and PSQI administration were completed on separate days; however, Aβ deposition is a relatively slow process, occurring over many years^7^, and sleep habits are usually chronic, particularly in the age group studied. Nevertheless, it is acknowledged that administration of the PSQI at multiple time points would provide a longer window of assessment of sleep parameters and therefore may be more informative. Finally, any inferences of potential glymphatic clearance underpinning the association of *AQP4* genetic variation with Aβ clearance from the brain in humans and the potential functional implications of genetic variation on isoform-specific expression are speculative and require further functional studies to elucidate.

Our study adds weight to the proposition that paravascular clearance, encompassing the postulated glymphatic system, is a potential biological mechanism that underpins Aβ clearance from the brain^14^. Whether other genetic factors beyond *APOE* and *AQP4*, examined here, may likewise moderate the relationship between sleep parameters and AD characteristics remains to be determined, however, the current study provides evidence to support future investigation of such interactions. Prospectively, the results of this study provide a greater understanding of what factors may impact on the sleep-AD phenotype relationship, and support the notion that establishing interventions targeted at improving sleep parameters maybe beneficial for positively modulating cerebral Aβ levels and thus, potentially delaying AD onset. Indeed, findings from this study could be used to both stratify retrospective analysis of existing datasets, or perhaps more importantly, to derive tailored AD intervention strategies based on the genetics of the individual: e.g. a sleep-specific intervention targeted at reducing sleep latency may be most beneficial to individuals who are genetically predisposed to a heightened impact of latency on pathological or clinical outcomes. Overall, the data from this study provide evidence that genetic variation in the cerebrally expressed water-channel protein, Aquaporin-4, moderates the relationship between sleep and brain Aβ burden.

## Electronic supplementary material

Supplementary Data
