# Supplementary material for: Genetic variation in Aquaporin-4 moderates the relationship between sleep and brain Aβ-amyloid burden
Source: Transl Psychiatry. 2018 Feb 26;8:47. doi: 10.1038/s41398-018-0094-x (PMC5865132; doi:10.1038/s41398-018-0094-x)
Supplement: Supplementary file 1 — Supplementary Data [file 41398_2018_94_MOESM1_ESM.docx]

**Supplementary Data**

**Supplementary Table 1: Complete SNP list for *AQP4* prior to LD pruning.**

| **SNP#** | **SNP Ref** | **Major Allele** | **Call Rate** | **Minor Allele** | **MAF** | **HWE-p** |
| --- | --- | --- | --- | --- | --- | --- |
| **1** | **rs11661081** | **C** | **1.00** | **A** | **0.09** | **0.89** |
| **2** | **rs9951307** | **A** | **1.00** | **G** | **0.36** | **0.65** |
| 3 | rs12455617 | C | 1.00 | A | 0.12 | 0.41 |
| 4 | rs16942851 | T | 0.99 | G | 0.19 | 0.98 |
| **5** | **rs7240333** | **C** | **0.99** | **T** | **0.11** | **0.41** |
| 6 | rs1058427 | G | 0.98 | T | 0.12 | 0.55 |
| 7 | rs14393 | G | 0.98 | T | 0.30 | 0.38 |
| 8 | rs1058424 | A | 0.98 | T | 0.18 | 0.59 |
| 9 | rs335929 | A | 1.00 | C | 0.19 | 0.98 |
| 10 | rs3763043 | C | 1.00 | T | 0.31 | 0.44 |
| **11** | **rs68006382** | **A** | **0.98** | **G** | **0.18** | **0.62** |
| 12 | rs335930 | A | 0.98 | C | 0.21 | 0.49 |
| 13 | rs11661256 | T | 0.98 | A | 0.12 | 0.49 |
| **14** | **rs71353406** | **C** | **0.98** | **A** | **0.30** | **0.45** |
| 15 | rs335931 | A | 0.98 | G | 0.19 | 0.97 |
| 16 | rs67207056 | G | 0.97 | A | 0.12 | 0.51 |
| 17 | rs55875625 | T | 0.97 | C | 0.12 | 0.49 |
| 18 | rs455671 | A | 0.97 | G | 0.19 | 1.00 |
| 19 | rs35248760 | C | 0.97 | A | 0.12 | 0.49 |
| **20** | **rs72878776** | **G** | **0.97** | **A** | **0.12** | **0.49** |
| 21 | rs63514 | C | 0.98 | T | 0.19 | 0.70 |
| 22 | rs12968026 | T | 0.97 | C | 0.12 | 0.48 |
| **23** | **rs3875089** | **T** | **1.00** | **C** | **0.16** | **0.84** |
| 24 | rs162008 | C | 0.99 | T | 0.19 | 0.93 |
| **25** | **rs162007** | **G** | **1.00** | **A** | **0.20** | **0.80** |
| **26** | **rs162003** | **C** | **1.00** | **T** | **0.08** | **0.86** |
| 27 | rs3834826 | - | 0.99 | C | 0.40 | 0.29 |
| 28 | rs11662318 | C | 0.98 | T | 0.16 | 0.67 |
| **29** | **rs151245** | **T** | **1.00** | **G** | **0.40** | **0.22** |
| **30** | **rs151246** | **G** | **0.99** | **T** | **0.20** | **0.11** |
| **31** | **rs2339214** | **G** | **0.98** | **A** | **0.48** | **0.63** |
| **32** | **rs491148** | **A** | **1.00** | **G** | **0.17** | **0.11** |

Final, post quality control, Aquaporin 4 (*AQP4*) single nucleotide polymorphisms (SNPs) selected for prior to Linkage Disequilibrium (LD) pruning. Final SNPs selected post LD-pruning are shown in bold. SNP Ref, the reference single nucleotide polymorphism (SNP) marker (rs). Exclusion criteria: Call rate < 95%; MAF, minor allele frequency < 5%; HWE-p, Hardy-Weinberg equilibrium p-value < 0.05. SNP#, Study allocated SNP number for cross-reference to Supplementary Figure 1.

**Supplementary Figure 1: Linkage disequilibrium (LD) structures for *AQP4***

LD structures *(a)* pre (32 SNPs), and *(b)* post (13 SNPs) LD pruning of Aquaporin-4 gene (*AQP4*)*.* LD pruning was undertaken in Golden Helix SVS (v8.7.1) using r^2^ > 0.8, window size 10, increment 5. Diamond graph: deep red indicates high r^2^ value, whilst deep blue indicates a lower r^2^ value. Vertical lines above the diamond graph indicate SNPs. SNP#, identifies the pre- and post-LD pruned SNPs as per Supplementary Table 1

**Supplementary Table 2: Association of *AQP4* genetic variants with PSQI Sleep Parameters.**

| **PSQI Sleep Parameter** | **SNP Ref** | **Additive**^†^ | | **Dominant**^†^ | | **Recessive**^†^ | |
| --- | --- | --- | --- | --- | --- | --- | --- |
|  |  | *Base** | *Adj** | *Base** | *Adj** | *Base** | *Adj** |
| PSQI Total | rs11661081 | 0.443 | 0.465 | 0.412 | 0.407 | 0.947 | 0.770 |
|  | rs9951307 | 0.730 | 0.794 | 0.836 | 0.627 | 0.297 | 0.189 |
|  | rs7240333 | 0.539 | 0.323 | 0.387 | 0.205 | 0.593 | 0.636 |
|  | rs68006382 | 0.284 | 0.360 | 0.251 | 0.348 | 0.779 | 0.722 |
|  | rs71353406 | 0.130 | 0.100 | **0.042** | **0.045** | 0.856 | 0.871 |
|  | rs12968026 | 0.593 | 0.836 | 0.647 | 0.466 | **0.0006†** | **0.002†** |
|  | rs3875089 | 0.494 | 0.442 | 0.940 | 0.931 | **0.012** | **0.021** |
|  | rs162007 | 0.761 | 0.545 | 0.968 | 0.723 | 0.374 | 0.338 |
|  | rs162003 | 0.926 | 0.802 | 0.747 | 0.999 | 0.307 | 0.226 |
|  | rs151245 | 0.698 | 0.697 | 0.928 | 0.787 | 0.396 | 0.277 |
|  | rs151246 | 0.572 | 0.636 | 0.407 | 0.366 | 0.715 | 0.424 |
|  | rs2339214 | 0.775 | 0.788 | 0.554 | 0.416 | 0.867 | 0.656 |
|  | rs491148 | 0.550 | 0.732 | 0.770 | 0.973 | 0.287 | 0.259 |
| Sleep Latency (minutes) | rs11661081 | 0.664 | 0.676 | 0.562 | 0.568 | 0.544 | 0.526 |
|  | rs9951307 | 0.777 | 0.756 | 0.883 | 0.984 | 0.721 | 0.536 |
|  | rs7240333 | 0.787 | 0.822 | 0.848 | 0.713 | 0.728 | 0.724 |
|  | rs68006382 | 0.171 | 0.170 | 0.189 | 0.182 | 0.456 | 0.470 |
|  | rs71353406 | 0.384 | 0.604 | 0.863 | 0.908 | 0.084 | 0.314 |
|  | rs12968026 | 0.283 | 0.340 | 0.277 | 0.372 | 0.708 | 0.571 |
|  | rs3875089 | 0.123 | 0.195 | 0.098 | 0.187 | 0.770 | 0.648 |
|  | rs162007 | 0.568 | 0.400 | 0.656 | 0.460 | 0.562 | 0.545 |
|  | rs162003 | 0.989 | 0.754 | 0.951 | 0.708 | 0.832 | 0.857 |
|  | rs151245 | 0.935 | 0.973 | 0.843 | 0.925 | 0.913 | 0.852 |
|  | rs151246 | 0.744 | 0.797 | 0.756 | 0.853 | 0.854 | 0.776 |
|  | rs2339214 | 0.876 | 0.630 | 0.925 | 0.744 | 0.868 | 0.636 |
|  | rs491148 | 0.484 | 0.583 | 0.594 | 0.732 | 0.457 | 0.438 |
| Sleep Duration (hours) | rs11661081 | 0.471 | 0.523 | 0.506 | 0.564 | 0.648 | 0.641 |
|  | rs9951307 | 0.722 | 0.551 | 0.555 | 0.695 | 0.096 | 0.062 |
|  | rs7240333 | 0.588 | 0.406 | 0.410 | 0.242 | 0.507 | 0.469 |
|  | rs68006382 | 0.991 | 0.750 | 0.824 | 0.867 | 0.546 | 0.601 |
|  | rs71353406 | 0.730 | 0.428 | 0.488 | 0.315 | 0.642 | 0.993 |
|  | rs12968026 | 0.411 | 0.482 | 0.163 | 0.204 | 0.124 | 0.126 |
|  | rs3875089 | 0.771 | 0.658 | 0.996 | 0.860 | 0.341 | 0.339 |
|  | rs162007 | 0.590 | 0.689 | 0.301 | 0.381 | 0.205 | 0.213 |
|  | rs162003 | 0.842 | 0.979 | 0.598 | 0.729 | 0.149 | 0.152 |
|  | rs151245 | 0.854 | 0.891 | 0.650 | 0.822 | 0.798 | 0.580 |
|  | rs151246 | 0.626 | 0.751 | 0.575 | 0.597 | 0.967 | 0.719 |
|  | rs2339214 | 0.769 | 0.693 | 0.795 | 0.726 | 0.827 | 0.767 |
|  | rs491148 | 0.510 | 0.623 | 0.530 | 0.662 | 0.713 | 0.720 |
| Sleep Disturbances | rs11661081 | 0.343 | 0.333 | 0.405 | 0.397 | 0.411 | 0.400 |
|  | rs9951307 | 0.563 | 0.418 | 0.627 | 0.653 | 0.660 | 0.326 |
|  | rs7240333 | 0.939 | 0.812 | 0.859 | 0.996 | 0.410 | 0.398 |
|  | rs68006382 | 0.097 | 0.146 | **0.034** | 0.077 | 0.672 | 0.902 |
|  | rs71353406 | 0.165 | 0.181 | 0.112 | 0.137 | 0.733 | 0.684 |
|  | rs12968026 | 0.886 | 0.548 | 0.848 | 0.576 | 0.919 | 0.716 |
|  | rs3875089 | 0.904 | 0.979 | 0.968 | 0.996 | 0.774 | 0.920 |
|  | rs162007 | 0.674 | 0.748 | 0.382 | 0.429 | 0.236 | 0.209 |
|  | rs162003 | 0.727 | 0.643 | 0.849 | 0.791 | 0.411 | 0.304 |
|  | rs151245 | 0.368 | 0.361 | 0.159 | 0.181 | 0.861 | 0.958 |
|  | rs151246 | 0.466 | 0.708 | 0.442 | 0.554 | 0.818 | 0.726 |
|  | rs2339214 | 0.140 | 0.093 | 0.287 | 0.126 | 0.171 | 0.226 |
|  | rs491148 | 0.076 | 0.188 | 0.084 | 0.206 | 0.382 | 0.483 |
| Sleep Efficiency | rs11661081 | 0.511 | 0.591 | 0.732 | 0.830 | 0.083 | 0.084 |
|  | rs9951307 | 0.308 | 0.325 | 0.272 | 0.289 | 0.693 | 0.708 |
|  | rs7240333 | 0.621 | 0.511 | 0.726 | 0.637 | 0.521 | 0.406 |
|  | rs68006382 | 0.275 | 0.268 | 0.186 | 0.207 | 0.917 | 0.907 |
|  | rs71353406 | 0.557 | 0.608 | 0.414 | 0.480 | 0.903 | 0.923 |
|  | rs12968026 | 0.708 | 0.519 | 0.799 | 0.637 | 0.593 | 0.421 |
|  | rs3875089 | 0.660 | 0.632 | 0.863 | 0.886 | 0.336 | 0.242 |
|  | rs162007 | 0.704 | 0.742 | 0.461 | 0.492 | 0.360 | 0.354 |
|  | rs162003 | 0.562 | 0.591 | 0.510 | 0.499 | 0.842 | 0.643 |
|  | rs151245 | 0.832 | 0.873 | 0.761 | 0.830 | 0.996 | 0.986 |
|  | rs151246 | 0.783 | 0.869 | 0.760 | 0.790 | 0.957 | 0.867 |
|  | rs2339214 | 0.906 | 0.946 | 0.827 | 0.946 | 0.968 | 0.845 |
|  | rs491148 | 0.663 | 0.887 | 0.510 | 0.701 | 0.687 | 0.571 |

PSQI, Pittsburgh Sleep Quality Index Sleep Parameters: PSQI total, Sleep Latency (minutes), Sleep Duration (hours), Sleep Disturbances, and Sleep Efficiency. SNP Ref, reference single nucleotide polymorphism marker (rs); *AQP4*, Aquaporin 4. †Genetic models: Additive (homozygote for the minor allele (MM) vs heterozygote for the minor allele (Mm) vs homozygote for the major allele (Mm)); Recessive (homozygote for the minor allele (MM) vs heterozygote/homozygote for the major allele (Mm/mm)); Dominant (heterozygote/homozygote for the minor allele (Mm or MM) vs homozygote for the major allele (mm)). *Statistical models: Base, base statistical model that is no covariates; Adj, Adjusted statistical model (covaries for: age, sex, body mass index (BMI), geriatric depression scale (GDS) and a medical history of CVD). Values that reached nominal significance (p < 0.05, uncorrected) are bolded. †values significant after False Discovery Rate correction (q < 0.05).

**Supplementary Table 3: Association of *AQP4* genetic variants with brain Aβ burden.**

| **SNP Ref** | **Additive**^†^ | | **Dominant**^†^ | | **Recessive**^†^ | |
| --- | --- | --- | --- | --- | --- | --- |
|  | ***Base**** | ***Adj**** | ***Base**** | ***Adj**** | ***Base**** | ***Adj**** |
| rs11661081 | 0.941 | 0.916 | 0.938 | 0.992 | 0.511 | 0.666 |
| rs9951307 | 0.194 | 0.153 | 0.220 | 0.147 | 0.441 | 0.489 |
| rs7240333 | 0.698 | 0.415 | 0.740 | 0.367 | 0.738 | 0.970 |
| rs68006382 | 0.510 | 0.329 | 0.521 | 0.394 | 0.744 | 0.450 |
| rs71353406 | 0.452 | 0.191 | 0.591 | 0.304 | 0.430 | 0.249 |
| rs72878776 | 0.471 | 0.389 | 0.246 | 0.298 | 0.405 | 0.935 |
| rs3875089 | 0.311 | 0.285 | 0.221 | 0.340 | 0.935 | 0.433 |
| rs162007 | 0.878 | 0.670 | 0.692 | 0.891 | 0.459 | 0.240 |
| rs162003 | 0.172 | 0.197 | 0.172 | 0.197 | - | - |
| rs151245 | 0.799 | 0.590 | 0.978 | 0.868 | 0.657 | 0.424 |
| rs151246 | 0.566 | 0.529 | 0.541 | 0.610 | 0.879 | 0.573 |
| rs2339214 | 0.393 | 0.662 | 0.728 | 0.964 | 0.288 | 0.441 |
| rs491148 | 0.261 | 0.098 | 0.273 | 0.195 | 0.537 | 0.099 |

SNP Ref, reference single nucleotide polymorphism marker (rs); *AQP4*, Aquaporin-4; MAF, Minor allele frequency; M, Minor allele; m, major allele; MM, homozygote for the minor allele; Mm, heterozygote for the minor allele; mm, homozygote for the major allele. †Genetic models: Additive (homozygote for the minor allele (MM) vs heterozygote for the minor allele (Mm) vs homozygote for the major allele (mm)); Recessive (homozygote for the minor allele (MM) vs heterozygote/homozygote for the major allele (Mm/mm)); Dominant (heterozygote/homozygote for the minor allele (Mm or MM) vs homozygote for the major allele (mm)). *Statistical models: Base, base statistical model including no covariates; Adj, Adjusted statistical model (covaries for: age, sex, Apolipoprotein E status (ε4-carrier/non-carrier).

**Supplementary Table 4: Linear regression analyses for the association of PSQI Sleep parameters with brain Aβ burden.**

| ***PSQI parameter*** | ***β*** | ***SE*** | ***t*** | ***Sig.*** | ***R^2^*** | ***F*** | ***Sig.*** |
| --- | --- | --- | --- | --- | --- | --- | --- |
| ***Model summary (PSQI Total)*** | |  |  |  | **0.134** | **5.526** | **<0.001** |
| PSQI total | 0.004 | 0.008 | 0.547 | 0.585 |  |  |  |
| Age | 0.011 | 0.004 | 2.513 | 0.013 |  |  |  |
| BMI | 0.004 | 0.006 | 0.730 | 0.466 |  |  |  |
| CVD risk | -0.011 | 0.040 | -0.269 | 0.788 |  |  |  |
| GDS | -0.010 | 0.016 | -0.617 | 0.538 |  |  |  |
| *APOE* ε4 | 0.305 | 0.058 | 5.265 | <0.001 |  |  |  |
| ***Model summary (Sleep Latency)*** | |  |  |  | **0.160** | **6.824** | **<0.001** |
| **Sleep latency** | **0.004** | **0.001** | **2.656** | **0.008** |  |  |  |
| Age | 0.010 | 0.004 | 2.499 | 0.013 |  |  |  |
| BMI | 0.004 | 0.006 | 0.741 | 0.459 |  |  |  |
| CVD risk | -0.012 | 0.039 | -0.314 | 0.754 |  |  |  |
| GDS | -0.010 | 0.015 | -0.684 | 0.495 |  |  |  |
| *APOE* ε4 | 0.306 | 0.057 | 5.375 | <0.001 |  |  |  |
| ***Model summary (Sleep Duration)*** | | |  |  | **0.133** | **5.483** | **<0.001** |
| Sleep duration | 0.006 | 0.020 | 0.276 | 0.783 |  |  |  |
| Age | 0.011 | 0.004 | 2.560 | 0.011 |  |  |  |
| BMI | 0.004 | 0.006 | 0.718 | 0.473 |  |  |  |
| CVD risk | -0.011 | 0.040 | -0.279 | 0.781 |  |  |  |
| GDS | -0.007 | 0.015 | -0.448 | 0.654 |  |  |  |
| *APOE* ε4 | 0.302 | 0.058 | 5.197 | <0.001 |  |  |  |
| ***Model summary (Sleep Disturbances)*** | | |  |  | **0.136** | **5.656** | **<0.001** |
| Sleep disturbances | -0.047 | 0.047 | -0.988 | 0.325 |  |  |  |
| Age | 0.010 | 0.004 | 2.520 | 0.012 |  |  |  |
| BMI | 0.004 | 0.006 | 0.631 | 0.528 |  |  |  |
| CVD risk | -0.004 | 0.040 | -0.091 | 0.927 |  |  |  |
| GDS | -0.005 | 0.015 | -0.333 | 0.740 |  |  |  |
| *APOE* ε4 | 0.304 | 0.058 | 5.254 | <0.001 |  |  |  |
| ***Model summary (Sleep Efficiency)*** | | |  |  | **0.135** | **5.599** | **<0.001** |
| Sleep efficiency | -0.027 | 0.033 | -0.822 | 0.412 |  |  |  |
| Age | 0.011 | 0.004 | 2.543 | 0.012 |  |  |  |
| BMI | 0.004 | 0.006 | 0.041 | 0.534 |  |  |  |
| CVD risk | -0.007 | 0.040 | -0.177 | 0.859 |  |  |  |
| GDS | -0.005 | 0.015 | -0.351 | 0.726 |  |  |  |
| *APOE* ε4 | 0.303 | 0.058 | 5.249 | <0.001 |  |  |  |
| ***Model summary (Daytime Dysfunction)*** | | |  |  | **0.111** | **5.584** | **<0.001** |
| Daytime dysfunction | 0.031 | 0.040 | 0.776 | 0.439 |  |  |  |
| Age | 0.010 | 0.004 | 2.446 | 0.015 |  |  |  |
| BMI | 0.004 | 0.006 | 0.730 | 0.466 |  |  |  |
| CVD risk | -0.014 | 0.040 | -0.345 | 0.731 |  |  |  |
| GDS | -0.012 | 0.016 | -0.742 | 0.459 |  |  |  |
| *APOE* ε4 | 0.304 | 0.058 | 5.250 | <0.001 |  |  |  |

PSQI, Pittsburgh Sleep Quality Index; β, coefficient of predictors; SE, standard error; t, Student’s t distribution test statistic; F, Fisher’s F ratio/ distribution; Sig, Significance (p-value); R^2^, coefficient of multiple determination; BMI, Body Mass Index; GDS, Geriatric Depression Scale; CVD risk, cardiovascular disease risk; *APOE* ε4, Apolipoprotein E ε4 allele carriage (presence/absence).

**Supplementary Table 5: Moderation Analysis for *AQP4* SNPs on PSQI total.**

|  |  |  | **Dominant**^†^ | |  |  |  |  | **Recessive**^†^ | |  |  |
| --- | --- | --- | --- | --- | --- | --- | --- | --- | --- | --- | --- | --- |
|  | **β** | **SE** | **Sig.** | **R^2^** | **Sig.** | **ΔR^2^** | **β** | **SE** | **Sig.** | **R^2^** | **Sig.** | **ΔR^2^** |
| *Model summary: rs11661081* | | |  | 0.136 | <0.001 |  |  |  |  | 0.135 | <0.001 |  |
| Age | 0.011 | 0.004 | 0.013 |  |  |  | 0.010 | 0.004 | 0.014 |  |  |  |
| BMI | 0.004 | 0.006 | 0.496 |  |  |  | 0.004 | 0.006 | 0.469 |  |  |  |
| CVD risk | -0.009 | 0.040 | 0.820 |  |  |  | -0.007 | 0.040 | 0.866 |  |  |  |
| GDS | -0.010 | 0.016 | 0.526 |  |  |  | -0.010 | 0.016 | 0.524 |  |  |  |
| APOE ε4 | 0.305 | 0.058 | <0.001 |  |  |  | 0.303 | 0.058 | <0.001 |  |  |  |
| rs11661081 | 0.084 | 0.142 | 0.554 |  |  |  | -0.691 | 1.820 | 0.704 |  |  |  |
| PSQI total | 0.007 | 0.009 | 0.449 |  |  |  | 0.005 | 0.008 | 0.579 |  |  |  |
| INT | -0.017 | 0.023 | 0.465 |  |  | 0.002 | 0.082 | 0.257 | 0.750 |  |  | <0.001 |
| *Model summary rs9951307* | | |  | 0.151 | <0.001 |  |  |  |  | 0.142 | <0.001 |  |
| Age | 0.010 | 0.004 | 0.015 |  |  |  | 0.011 | 0.004 | 0.011 |  |  |  |
| BMI | 0.005 | 0.006 | 0.423 |  |  |  | 0.006 | 0.006 | 0.362 |  |  |  |
| CVD risk | -0.009 | 0.039 | 0.818 |  |  |  | -0.007 | 0.040 | 0.863 |  |  |  |
| GDS | -0.008 | 0.016 | 0.636 |  |  |  | -0.011 | 0.016 | 0.490 |  |  |  |
| APOE ε4 | 0.306 | 0.058 | <0.001 |  |  |  | 0.301 | 0.058 | <0.001 |  |  |  |
| rs9951307 | -0.200 | 0.101 | 0.049 |  |  |  | -0.241 | 0.177 | 0.174 |  |  |  |
| PSQI total | -0.010 | 0.013 | 0.480 |  |  |  | 0.002 | 0.009 | 0.780 |  |  |  |
| INT | 0.022 | 0.016 | 0.172 |  |  | 0.008 | 0.025 | 0.024 | 0.286 |  |  | 0.005 |
| *Model summary: rs7240333* | | |  | 0.151 | <0.001 |  |  |  |  | 0.152 | <0.001 |  |
| Age | 0.010 | 0.004 | 0.016 |  |  |  | 0.011 | 0.004 | 0.011 |  |  |  |
| BMI | 0.004 | 0.006 | 0.518 |  |  |  | 0.004 | 0.006 | 0.528 |  |  |  |
| CVD risk | -0.004 | 0.040 | 0.915 |  |  |  | -0.003 | 0.040 | 0.947 |  |  |  |
| GDS | -0.012 | 0.016 | 0.441 |  |  |  | -0.014 | 0.016 | 0.376 |  |  |  |
| APOE ε4 | 0.329 | 0.059 | <0.001 |  |  |  | 0.320 | 0.058 | <0.001 |  |  |  |
| rs7240333 | 0.025 | 0.125 | 0.842 |  |  |  | 0.252 | 0.351 | 0.473 |  |  |  |
| PSQI total | 0.005 | 0.009 | 0.562 |  |  |  | 0.007 | 0.008 | 0.390 |  |  |  |
| INT | 0.005 | 0.020 | 0.823 |  |  | <0.001 | -0.050 | 0.053 | 0.348 |  |  |  |

*(continued over)*

**Supplementary Table 5: Moderation Analysis for *AQP4* SNPs on PSQI total (*cont)*.**

|  |  |  | **Dominant**^†^ | |  |  |  |  | **Recessive**^†^ | |  |  |
| --- | --- | --- | --- | --- | --- | --- | --- | --- | --- | --- | --- | --- |
|  | **β** | **SE** | **Sig.** | **R^2^** | **Sig.** | **ΔR^2^** | **β** | **SE** | **Sig.** | **R^2^** | **Sig.** | **ΔR^2^** |
| *Model summary: rs68006382* | | |  | 0.140 | <0.001 |  |  |  |  | 0.148 | <0.001 |  |
| Age | 0.011 | 0.004 | 0.012 |  |  |  | 0.010 | 0.004 | 0.019 |  |  |  |
| BMI | 0.006 | 0.006 | 0.340 |  |  |  | 0.005 | 0.006 | 0.409 |  |  |  |
| CVD risk | -0.005 | 0.040 | 0.903 |  |  |  | -0.009 | 0.040 | 0.823 |  |  |  |
| GDS | -0.009 | 0.016 | 0.574 |  |  |  | -0.008 | 0.016 | 0.624 |  |  |  |
| APOE ε4 | 0.302 | 0.059 | <0.001 |  |  |  | 0.307 | 0.059 | <0.001 |  |  |  |
| rs68006382 | 0.040 | 0.113 | 0.725 |  |  |  | 0.475 | 0.304 | 0.119 |  |  |  |
| PSQI total | 0.006 | 0.009 | 0.528 |  |  |  | 0.007 | 0.008 | 0.396 |  |  |  |
| INT | 0.001 | 0.019 | 0.978 |  |  | <0.001 | -0.082 | 0.061 | 0.179 |  |  | 0.008 |
| *Model summary: rs71353406* | | |  | 0.136 | <0.001 |  |  |  |  | 0.150 | <0.001 |  |
| Age | 0.010 | 0.004 | 0.017 |  |  |  | 0.010 | 0.004 | 0.023 |  |  |  |
| BMI | 0.005 | 0.006 | 0.397 |  |  |  | 0.005 | 0.006 | 0.398 |  |  |  |
| CVD risk | -0.010 | 0.040 | 0.796 |  |  |  | -0.014 | 0.040 | 0.727 |  |  |  |
| GDS | -0.009 | 0.016 | 0.601 |  |  |  | -0.011 | 0.016 | 0.512 |  |  |  |
| APOE ε4 | 0.303 | 0.059 | <0.001 |  |  |  | 0.303 | 0.059 | <0.001 |  |  |  |
| rs71353406 | 0.026 | 0.101 | 0.796 |  |  |  | 0.450 | 0.212 | 0.035 |  |  |  |
| PSQI total | 0.004 | 0.011 | 0.682 |  |  |  | 0.009 | 0.008 | 0.294 |  |  |  |
| INT | 0.004 | 0.016 | 0.822 |  |  | <0.001 | -0.065 | 0.036 | 0.073 |  |  | 0.013 |
| *Model summary: rs12968026* | | |  | 0.136 | <0.001 |  |  |  |  | 0.128 | <0.001 |  |
| Age | 0.011 | 0.004 | 0.008 |  |  |  | 0.011 | 0.004 | 0.013 |  |  |  |
| BMI | 0.005 | 0.006 | 0.419 |  |  |  | 0.004 | 0.006 | 0.511 |  |  |  |
| CVD risk | -0.016 | 0.041 | 0.699 |  |  |  | -0.009 | 0.040 | 0.827 |  |  |  |
| GDS | -0.009 | 0.016 | 0.584 |  |  |  | -0.007 | 0.016 | 0.650 |  |  |  |
| APOE ε4 | 0.283 | 0.058 | <0.001 |  |  |  | 0.284 | 0.059 | <0.001 |  |  |  |
| rs12968026 | -0.003 | 0.116 | 0.981 |  |  |  | -0.112 | 0.464 | 0.810 |  |  |  |
| PSQI total | 0.003 | 0.009 | 0.738 |  |  |  | 0.006 | 0.008 | 0.453 |  |  |  |
| INT | 0.013 | 0.018 | 0.452 |  |  | 0.002 | 0.009 | 0.045 | 0.846 |  |  | <0.001 |

*(continued over)*

**Supplementary Table 5: Moderation Analysis for *AQP4* SNPs on PSQI total (*cont)*.**

|  |  |  | **Dominant**^†^ | |  |  |  |  | **Recessive**^†^ | |  |  |
| --- | --- | --- | --- | --- | --- | --- | --- | --- | --- | --- | --- | --- |
|  | **β** | **SE** | **Sig.** | **R^2^** | **Sig.** | **ΔR^2^** | **β** | **SE** | **Sig.** | **R^2^** | **Sig.** | **ΔR^2^** |
| *Model summary: rs3875089* | | |  | 0.138 | <0.001 |  |  |  |  | 0.142 | <0.001 |  |
| Age | 0.011 | 0.004 | 0.013 |  |  |  | 0.011 | 0.004 | 0.013 |  |  |  |
| BMI | 0.005 | 0.006 | 0.445 |  |  |  | 0.005 | 0.006 | 0.440 |  |  |  |
| CVD risk | -0.010 | 0.040 | 0.812 |  |  |  | -0.010 | 0.040 | 0.805 |  |  |  |
| GDS | -0.010 | 0.016 | 0.527 |  |  |  | -0.009 | 0.016 | 0.591 |  |  |  |
| APOE ε4 | 0.300 | 0.058 | <0.001 |  |  |  | 0.309 | 0.058 | <0.001 |  |  |  |
| rs3875089 | 0.044 | 0.104 | 0.673 |  |  |  | 0.457 | 0.317 | 0.151 |  |  |  |
| PSQI total | 0.004 | 0.010 | 0.678 |  |  |  | 0.005 | 0.008 | 0.518 |  |  |  |
| INT | 0.002 | 0.016 | 0.893 |  |  | <0.001 | -0.041 | 0.035 | 0.235 |  |  | 0.006 |
| *Model summary: rs162007* | | |  | 0.134 | <0.001 |  |  |  |  | 0.135 | <0.001 |  |
| Age | 0.010 | 0.004 | 0.014 |  |  |  | 0.010 | 0.004 | 0.016 |  |  |  |
| BMI | 0.004 | 0.006 | 0.480 |  |  |  | 0.004 | 0.006 | 0.473 |  |  |  |
| CVD risk | -0.010 | 0.040 | 0.807 |  |  |  | -0.011 | 0.040 | 0.790 |  |  |  |
| GDS | -0.010 | 0.016 | 0.543 |  |  |  | -0.011 | 0.016 | 0.511 |  |  |  |
| APOE ε4 | 0.305 | 0.058 | <0.001 |  |  |  | 0.306 | 0.058 | <0.001 |  |  |  |
| rs162007 | -0.020 | 0.105 | 0.847 |  |  |  | -0.264 | 0.608 | 0.665 |  |  |  |
| PSQI total | 0.003 | 0.010 | 0.737 |  |  |  | 0.005 | 0.008 | 0.574 |  |  |  |
| INT | 0.004 | 0.017 | 0.819 |  |  | <0.001 | 0.027 | 0.089 | 0.762 |  |  | <0.001 |
| *Model summary: rs162003* | | |  | 0.138 | <0.001 |  |  |  |  | N/A |  |  |
| Age | 0.010 | 0.004 | 0.015 |  |  |  |  |  |  |  |  |  |
| BMI | 0.004 | 0.006 | 0.555 |  |  |  |  |  |  |  |  |  |
| CVD risk | -0.009 | 0.040 | 0.821 |  |  |  |  |  |  |  |  |  |
| GDS | -0.010 | 0.016 | 0.544 |  |  |  |  |  |  |  |  |  |
| APOE ε4 | 0.303 | 0.058 | <0.001 |  |  |  |  |  |  |  |  |  |
| rs162003 | -0.122 | 0.137 | 0.374 |  |  |  |  |  |  |  |  |  |
| PSQI total | 0.002 | 0.009 | 0.783 |  |  |  |  |  |  |  |  |  |
| INT | 0.011 | 0.024 | 0.650 |  |  | 0.001 |  |  |  |  |  |  |

*(continued over)*

**Supplementary Table 5: Moderation Analysis for *AQP4* SNPs on PSQI total (*cont)*.**

|  |  |  | **Dominant**^†^ | |  |  |  |  | **Recessive**^†^ | |  |  |
| --- | --- | --- | --- | --- | --- | --- | --- | --- | --- | --- | --- | --- |
|  | **β** | **SE** | **Sig.** | **R^2^** | **Sig.** | **ΔR^2^** | **β** | **SE** | **Sig.** | **R^2^** | **Sig.** | **ΔR^2^** |
| *Model summary: rs151245* | | |  | 0.134 | <0.001 |  |  |  |  | 0.150 | <0.001 |  |
| Age | 0.011 | 0.004 | 0.014 |  |  |  | 0.010 | 0.004 | 0.017 |  |  |  |
| BMI | 0.004 | 0.006 | 0.468 |  |  |  | 0.004 | 0.006 | 0.504 |  |  |  |
| CVD risk | -0.011 | 0.040 | 0.791 |  |  |  | -0.004 | 0.040 | 0.916 |  |  |  |
| GDS | -0.010 | 0.016 | 0.541 |  |  |  | -0.011 | 0.016 | 0.477 |  |  |  |
| APOE ε4 | 0.304 | 0.058 | <0.001 |  |  |  | 0.310 | 0.058 | <0.001 |  |  |  |
| rs151245 | 0.008 | 0.105 | 0.943 |  |  |  | 0.263 | 0.132 | 0.048 |  |  |  |
| PSQI total | 0.005 | 0.014 | 0.713 |  |  |  | 0.011 | 0.009 | 0.212 |  |  |  |
| INT | -0.001 | 0.017 | 0.944 |  |  | <0.001 | -0.035 | 0.019 | 0.070 |  |  | 0.013 |
| *Model summary: rs151246* | | |  | 0.148 | <0.001 |  |  |  |  | 0.141 | <0.001 |  |
| Age | 0.011 | 0.004 | 0.010 |  |  |  | 0.011 | 0.004 | 0.011 |  |  |  |
| BMI | 0.005 | 0.006 | 0.369 |  |  |  | 0.004 | 0.006 | 0.465 |  |  |  |
| CVD risk | -0.018 | 0.040 | 0.648 |  |  |  | -0.012 | 0.040 | 0.767 |  |  |  |
| GDS | -0.013 | 0.016 | 0.416 |  |  |  | -0.010 | 0.016 | 0.525 |  |  |  |
| APOE ε4 | 0.305 | 0.058 | <0.001 |  |  |  | 0.308 | 0.058 | <0.001 |  |  |  |
| rs151246 | 0.124 | 0.102 | 0.224 |  |  |  | 0.223 | 0.269 | 0.408 |  |  |  |
| PSQI total | 0.016 | 0.010 | 0.122 |  |  |  | 0.006 | 0.008 | 0.461 |  |  |  |
| INT | -0.028 | 0.016 | 0.079 |  |  | 0.013 | -0.050 | 0.041 | 0.226 |  |  | 0.006 |
| *Model summary: rs2339214* | | |  | 0.140 | <0.001 |  |  |  |  | 0.194 | <0.001 |  |
| Age | 0.010 | 0.004 | 0.028 |  |  |  | 0.010 | 0.004 | 0.019 |  |  |  |
| BMI | 0.005 | 0.006 | 0.440 |  |  |  | 0.005 | 0.006 | 0.442 |  |  |  |
| CVD risk | -0.007 | 0.040 | 0.854 |  |  |  | -0.007 | 0.041 | 0.863 |  |  |  |
| GDS | -0.008 | 0.016 | 0.633 |  |  |  | -0.007 | 0.016 | 0.666 |  |  |  |
| APOE ε4 | 0.303 | 0.059 | <0.001 |  |  |  | 0.303 | 0.059 | <0.001 |  |  |  |
| rs2339214 | -0.136 | 0.111 | 0.221 |  |  |  | 0.194 | 0.125 | 0.123 |  |  |  |
| PSQI total | -0.012 | 0.014 | 0.395 |  |  |  | 0.009 | 0.009 | 0.344 |  |  |  |
| INT | 0.023 | 0.017 | 0.170 |  |  | 0.008 | -0.027 | 0.019 | 0.169 |  |  | 0.008 |

*(continued over)*

**Supplementary Table 5: Moderation Analysis for *AQP4* SNPs on PSQI total (*cont)*.**

|  |  |  | **Dominant**^†^ | |  |  |  |  | **Recessive**^†^ | |  |  |
| --- | --- | --- | --- | --- | --- | --- | --- | --- | --- | --- | --- | --- |
|  | **β** | **SE** | **Sig.** | **R^2^** | **Sig.** | **ΔR^2^** | **β** | **SE** | **Sig.** | **R^2^** | **Sig.** | **ΔR^2^** |
| *Model summary: rs491148* | | |  | 0.144 | <0.001 |  |  |  |  | 0.148 | <0.001 |  |
| Age | 0.011 | 0.004 | 0.009 |  |  |  | 0.012 | 0.004 | 0.008 |  |  |  |
| BMI | 0.005 | 0.006 | 0.387 |  |  |  | 0.005 | 0.006 | 0.448 |  |  |  |
| CVD risk | -0.020 | 0.041 | 0.624 |  |  |  | -0.013 | 0.040 | 0.746 |  |  |  |
| GDS | -0.014 | 0.016 | 0.396 |  |  |  | -0.009 | 0.016 | 0.575 |  |  |  |
| APOE ε4 | 0.310 | 0.058 | <0.001 |  |  |  | 0.316 | 0.058 | <0.001 |  |  |  |
| rs491148 | -0.010 | 0.108 | 0.926 |  |  |  | 0.417 | 0.279 | 0.137 |  |  |  |
| PSQI total | 0.001 | 0.010 | 0.968 |  |  |  | 0.004 | 0.008 | 0.645 |  |  |  |
| INT | 0.015 | 0.017 | 0.372 |  |  | 0.003 | -0.027 | 0.033 | 0.415 |  |  | 0.003 |

Model summary statistics for all Aquaporin 4 (*AQP4*) reference single nucleotide polymorphism (SNP) markers (rs). †Genetic models: Dominant (heterozygote/homozygote for the minor allele (Mm or MM) vs homozygote for the major allele (mm)), Recessive (homozygote for the minor allele (MM) vs heterozygote/homozygote for the major allele (Mm/mm)); β, Coefficient of predictors: SE, standard error; Sig, p-value; R^2^, coefficient of multiple determination, ΔR^2^, multiple correlation coefficient (R) squared change; BMI, Body Mass Index; CVD risk, cardiovascular disease risk; GDS, Geriatric Depression Scale; APOE, Apolipoprotein E ε4 allele carriage (presence/absence); INT, Interaction (PSQI total * model summary SNP). Models where the interaction term (INT) resulted in a statistically significant R^2^-change (p < 0.05) are indicated (bolded). If a model could not run due to that SNP variable being a constant then not applicable (N/A) is recorded for that model.

**Supplementary Table 6: Moderation Analysis for *AQP4* SNPs on Sleep Latency.**

|  |  |  | **Dominant**^†^ | |  |  |  |  | **Recessive**^†^ | |  |  |
| --- | --- | --- | --- | --- | --- | --- | --- | --- | --- | --- | --- | --- |
|  | **β** | **SE** | **Sig.** | **R^2^** | **Sig.** | **ΔR^2^** | **β** | **SE** | **Sig.** | **R^2^** | **Sig.** | **ΔR^2^** |
| *Model summary: rs11661081* | | |  | 0.169 | <0.001 |  |  |  |  | 0.162 | <0.001 |  |
| Age | 0.010 | 0.004 | 0.019 |  |  |  | 0.010 | 0.004 | 0.015 |  |  |  |
| BMI | 0.004 | 0.006 | 0.502 |  |  |  | 0.004 | 0.006 | 0.467 |  |  |  |
| CVD risk | -0.011 | 0.039 | 0.785 |  |  |  | -0.008 | 0.040 | 0.839 |  |  |  |
| GDS | -0.010 | 0.015 | 0.504 |  |  |  | -0.011 | 0.015 | 0.466 |  |  |  |
| APOE ε4 | 0.307 | 0.057 | <0.001 |  |  |  | 0.304 | 0.057 | <0.001 |  |  |  |
| rs11661081 | 0.111 | 0.099 | 0.262 |  |  |  | -0.144 | 0.397 | 0.717 |  |  |  |
| Latency | 0.005 | 0.002 | 0.003 |  |  |  | 0.004 | 0.002 | 0.008 |  |  |  |
| INT | -0.007 | 0.005 | 0.132 |  |  | 0.009 | -0.001 | 0.008 | 0.927 |  |  | <0.001 |
| *Model summary rs9951307* | | |  | 0.186 | <0.001 |  |  |  |  | 0.166 | <0.001 |  |
| Age | 0.010 | 0.004 | 0.019 |  |  |  | 0.010 | 0.004 | 0.013 |  |  |  |
| BMI | 0.003 | 0.006 | 0.607 |  |  |  | 0.004 | 0.006 | 0.752 |  |  |  |
| CVD risk | -0.013 | 0.039 | 0.741 |  |  |  | -0.012 | 0.039 | 0.752 |  |  |  |
| GDS | -0.007 | 0.015 | 0.658 |  |  |  | -0.010 | 0.015 | 0.485 |  |  |  |
| *APOE* ε4 | 0.312 | 0.056 | <0.001 |  |  |  | 0.309 | 0.057 | <0.001 |  |  |  |
| rs9951307 | 0.015 | 0.070 | 0.831 |  |  |  | 0.025 | 0.123 | 0.837 |  |  |  |
| Latency | 0.008 | 0.002 | 0.001 |  |  |  | 0.004 | 0.002 | 0.006 |  |  |  |
| INT | **-0.006** | **0.003** | **0.048** |  |  | **0.015** | -0.005 | 0.006 | 0.347 |  |  | 0.004 |
| *Model summary: rs7240333* | | |  | 0.184 | <0.001 |  |  |  |  | 0.182 | <0.001 |  |
| Age | 0.010 | 0.004 | 0.015 |  |  |  | 0.011 | 0.004 | 0.010 |  |  |  |
| BMI | 0.004 | 0.006 | 0.554 |  |  |  | 0.004 | 0.006 | 0.518 |  |  |  |
| CVD risk | -0.008 | 0.039 | 0.845 |  |  |  | -0.004 | 0.039 | 0.921 |  |  |  |
| GDS | -0.011 | 0.015 | 0.473 |  |  |  | -0.013 | 0.015 | 0.378 |  |  |  |
| APOE ε4 | 0.328 | 0.058 | <0.001 |  |  |  | 0.319 | 0.057 | <0.001 |  |  |  |
| rs7240333 | -0.027 | 0.081 | 0.736 |  |  |  | 0.251 | 0.297 | 0.398 |  |  |  |
| Latency | 0.003 | 0.002 | 0.111 |  |  |  | 0.004 | 0.001 | 0.005 |  |  |  |
| INT | 0.004 | 0.003 | 0.218 |  |  | 0.006 | -0.016 | 0.013 | 0.225 |  |  | 0.006 |

*(continued over)*

**Supplementary Table 6: Moderation Analysis for *AQP4* SNPs on Sleep Latency (*cont)*.**

|  |  |  | **Dominant**^†^ | |  |  |  |  | **Recessive**^†^ | |  |  |
| --- | --- | --- | --- | --- | --- | --- | --- | --- | --- | --- | --- | --- |
|  | **β** | **SE** | **Sig.** | **R^2^** | **Sig.** | **ΔR^2^** | **β** | **SE** | **Sig.** | **R^2^** | **Sig.** | **ΔR^2^** |
| *Model summary: rs68006382* | | |  | 0.174 | <0.001 |  |  |  |  | 0.178 | <0.001 |  |
| Age | 0.011 | 0.004 | 0.012 |  |  |  | 0.010 | 0.004 | 0.017 |  |  |  |
| BMI | 0.005 | 0.006 | 0.406 |  |  |  | 0.005 | 0.006 | 0.460 |  |  |  |
| CVD risk | -0.001 | 0.040 | 0.980 |  |  |  | -0.008 | 0.040 | 0.847 |  |  |  |
| GDS | -0.008 | 0.015 | 0.582 |  |  |  | -0.009 | 0.015 | 0.536 |  |  |  |
| APOE ε4 | 0.301 | 0.058 | <0.001 |  |  |  | 0.309 | 0.058 | <0.001 |  |  |  |
| rs68006382 | -0.036 | 0.083 | 0.667 |  |  |  | -0.113 | 0.203 | 0.578 |  |  |  |
| Latency | 0.003 | 0.002 | 0.062 |  |  |  | 0.004 | 0.002 | 0.019 |  |  |  |
| INT | 0.004 | 0.004 | 0.231 |  |  | 0.006 | 0.013 | 0.008 | 0.108 |  |  | 0.011 |
| *Model summary: rs71353406* | | |  | 0.180 | <0.001 |  |  |  |  | 0.163 | <0.001 |  |
| Age | 0.010 | 0.004 | 0.023 |  |  |  | 0.010 | 0.004 | 0.018 |  |  |  |
| BMI | 0.004 | 0.006 | 0.556 |  |  |  | 0.005 | 0.006 | 0.401 |  |  |  |
| CVD risk | -0.008 | 0.039 | 0.833 |  |  |  | -0.012 | 0.040 | 0.769 |  |  |  |
| GDS | -0.006 | 0.015 | 0.692 |  |  |  | -0.009 | 0.015 | 0.552 |  |  |  |
| *APOE* ε4 | 0.298 | 0.058 | <0.001 |  |  |  | 0.307 | 0.058 | <0.001 |  |  |  |
| rs71353406 | -0.063 | 0.069 | 0.362 |  |  |  | 0.050 | 0.158 | 0.754 |  |  |  |
| Latency | 0.001 | 0.002 | 0.688 |  |  |  | 0.004 | 0.002 | 0.022 |  |  |  |
| INT | **0.006** | **0.003** | **0.030** |  |  | **0.019** | 0.003 | 0.006 | 0.675 |  |  | 0.001 |
| *Model summary: rs12968026* | | |  | 0.176 | <0.001 |  |  |  |  | 0.157 | <0.001 |  |
| Age | 0.010 | 0.004 | 0.011 |  |  |  | 0.011 | 0.004 | 0.012 |  |  |  |
| BMI | 0.005 | 0.006 | 0.414 |  |  |  | 0.004 | 0.006 | 0.515 |  |  |  |
| CVD risk | -0.016 | 0.039 | 0.683 |  |  |  | -0.009 | 0.039 | 0.816 |  |  |  |
| GDS | -0.006 | 0.015 | 0.687 |  |  |  | -0.007 | 0.015 | 0.647 |  |  |  |
| APOE ε4 | 0.287 | 0.057 | <0.001 |  |  |  | 0.287 | 0.058 | <0.001 |  |  |  |
| rs12968026 | -0.041 | 0.083 | 0.620 |  |  |  | -0.145 | 0.432 | 0.738 |  |  |  |
| Latency | 0.002 | 0.002 | 0.145 |  |  |  | 0.004 | 0.001 | 0.007 |  |  |  |
| INT | 0.006 | 0.003 | 0.056 |  |  | 0.015 | 0.012 | 0.029 | 0.687 |  |  | 0.001 |

*(continued over)*

**Supplementary Table 6: Moderation Analysis for *AQP4* SNPs on Sleep Latency (*cont)*.**

|  |  |  | **Dominant**^†^ | |  |  |  |  | **Recessive**^†^ | |  |  |
| --- | --- | --- | --- | --- | --- | --- | --- | --- | --- | --- | --- | --- |
|  | **β** | **SE** | **Sig.** | **R^2^** | **Sig.** | **ΔR^2^** | **β** | **SE** | **Sig.** | **R^2^** | **Sig.** | **ΔR^2^** |
| *Model summary: rs3875089* | | |  | 0.184 | <0.001 |  |  |  |  | 0.165 | <0.001 |  |
| Age | 0.010 | 0.004 | 0.019 |  |  |  | 0.011 | 0.004 | 0.010 |  |  |  |
| BMI | 0.005 | 0.006 | 0.400 |  |  |  | 0.004 | 0.006 | 0.458 |  |  |  |
| CVD risk | -0.016 | 0.039 | 0.683 |  |  |  | -0.017 | 0.040 | 0.660 |  |  |  |
| GDS | -0.010 | 0.015 | 0.501 |  |  |  | -0.012 | 0.015 | 0.426 |  |  |  |
| *APOE* ε4 | 0.310 | 0.057 | <0.001 |  |  |  | 0.313 | 0.058 | <0.001 |  |  |  |
| rs3875089 | -0.050 | 0.074 | 0.497 |  |  |  | -0.005 | 0.416 | 0.990 |  |  |  |
| Latency | 0.002 | 0.002 | 0.248 |  |  |  | 0.004 | 0.002 | 0.008 |  |  |  |
| INT | **0.007** | **0.003** | **0.028** |  |  | **0.019** | 0.010 | 0.027 | 0.706 |  |  | 0.001 |
| *Model summary: rs162007* | | |  | 0.163 | <0.001 |  |  |  |  | 0.161 | <0.001 |  |
| Age | 0.010 | 0.004 | 0.015 |  |  |  | 0.010 | 0.004 | 0.015 |  |  |  |
| BMI | 0.004 | 0.006 | 0.481 |  |  |  | 0.004 | 0.006 | 0.464 |  |  |  |
| CVD risk | -0.012 | 0.039 | 0.753 |  |  |  | -0.013 | 0.039 | 0.745 |  |  |  |
| GDS | -0.009 | 0.015 | 0.557 |  |  |  | -0.010 | 0.015 | 0.498 |  |  |  |
| APOE ε4 | 0.307 | 0.057 | <0.001 |  |  |  | 0.306 | 0.057 | <0.001 |  |  |  |
| rs162007 | 0.041 | 0.071 | 0.564 |  |  |  | -0.071 | 0.263 | 0.786 |  |  |  |
| Latency | 0.005 | 0.002 | 0.010 |  |  |  | 0.004 | 0.002 | 0.010 |  |  |  |
| INT | -0.003 | 0.003 | 0.364 |  |  | 0.003 | 0.001 | 0.018 | 0.963 |  |  | <0.001 |
| *Model summary: rs162003* | | |  | 0.168 | <0.001 |  |  |  |  | N/A |  |  |
| Age | 0.009 | 0.004 | 0.024 |  |  |  |  |  |  |  |  |  |
| BMI | 0.003 | 0.006 | 0.560 |  |  |  |  |  |  |  |  |  |
| CVD risk | -0.011 | 0.039 | 0.784 |  |  |  |  |  |  |  |  |  |
| GDS | -0.009 | 0.015 | 0.558 |  |  |  |  |  |  |  |  |  |
| APOE ε4 | 0.302 | 0.057 | <0.001 |  |  |  |  |  |  |  |  |  |
| rs162003 | -0.007 | 0.092 | 0.941 |  |  |  |  |  |  |  |  |  |
| Latency | 0.005 | 0.092 | 0.941 |  |  |  |  |  |  |  |  |  |
| INT | -0.004 | 0.004 | 0.286 |  |  | 0.005 |  |  |  |  |  |  |

*(continued over)*

**Supplementary Table 6: Moderation Analysis for *AQP4* SNPs on Sleep Latency (*cont)*.**

|  |  |  | **Dominant**^†^ | |  |  |  |  | **Recessive**^†^ | |  |  |
| --- | --- | --- | --- | --- | --- | --- | --- | --- | --- | --- | --- | --- |
|  | **β** | **SE** | **Sig.** | **R^2^** | **Sig.** | **ΔR^2^** | **β** | **SE** | **Sig.** | **R^2^** | **Sig.** | **ΔR^2^** |
| *Model summary: rs151245* | | |  | 0.165 | <0.001 |  |  |  |  | 0.163 | <0.001 |  |
| Age | 0.010 | 0.004 | 0.013 |  |  |  | 0.011 | 0.004 | 0.011 |  |  |  |
| BMI | 0.004 | 0.006 | 0.498 |  |  |  | 0.004 | 0.006 | 0.504 |  |  |  |
| CVD risk | -0.012 | 0.039 | 0.763 |  |  |  | -0.012 | 0.039 | 0.754 |  |  |  |
| GDS | -0.009 | 0.015 | 0.543 |  |  |  | -0.012 | 0.015 | 0.439 |  |  |  |
| APOE ε4 | 0.300 | 0.057 | <0.001 |  |  |  | 0.308 | 0.057 | <0.001 |  |  |  |
| rs151245 | 0.065 | 0.079 | 0.414 |  |  |  | 0.069 | 0.107 | 0.524 |  |  |  |
| Latency | 0.007 | 0.003 | 0.032 |  |  |  | 0.004 | 0.002 | 0.011 |  |  |  |
| INT | -0.004 | 0.004 | 0.275 |  |  | 0.005 | -0.001 | 0.005 | 0.928 |  |  | <0.001 |
| *Model summary: rs151246* | | |  | 0.201 | <0.001 |  |  |  |  | 0.165 | <0.001 |  |
| Age | 0.009 | 0.004 | 0.023 |  |  |  | 0.011 | 0.004 | 0.012 |  |  |  |
| BMI | 0.004 | 0.006 | 0.525 |  |  |  | 0.005 | 0.006 | 0.448 |  |  |  |
| CVD risk | -0.015 | 0.038 | 0.699 |  |  |  | -0.014 | 0.039 | 0.731 |  |  |  |
| GDS | -0.007 | 0.015 | 0.654 |  |  |  | -0.011 | 0.015 | 0.469 |  |  |  |
| *APOE* ε4 | 0.303 | 0.056 | <0.001 |  |  |  | 0.310 | 0.057 | <0.001 |  |  |  |
| rs151246 | 0.117 | 0.070 | 0.096 |  |  |  | 0.064 | 0.175 | 0.716 |  |  |  |
| Latency | 0.009 | 0.002 | <0.001 |  |  |  | 0.004 | 0.002 | 0.006 |  |  |  |
| INT | **-0.009** | **0.003** | **0.002** |  |  | **0.039** | -0.008 | 0.007 | 0.294 |  |  | 0.004 |
| *Model summary: rs2339214* | | |  | 0.164 | <0.001 |  |  |  |  | 0.165 | <0.001 |  |
| Age | 0.010 | 0.004 | 0.024 |  |  |  | 0.010 | 0.004 | 0.023 |  |  |  |
| BMI | 0.005 | 0.006 | 0.435 |  |  |  | 0.005 | 0.006 | 0.423 |  |  |  |
| CVD risk | -0.012 | 0.040 | 0.768 |  |  |  | -0.005 | 0.040 | 0.896 |  |  |  |
| GDS | -0.007 | 0.015 | 0.638 |  |  |  | -0.007 | 0.015 | 0.642 |  |  |  |
| APOE ε4 | 0.306 | 0.058 | <0.001 |  |  |  | 0.301 | 0.058 | <0.001 |  |  |  |
| rs2339214 | -0.059 | 0.078 | 0.451 |  |  |  | -0.029 | 0.092 | 0.758 |  |  |  |
| Latency | 0.002 | 0.003 | 0.506 |  |  |  | 0.003 | 0.002 | 0.039 |  |  |  |
| INT | 0.003 | 0.003 | 0.342 |  |  | 0.004 | 0.004 | 0.004 | 0.368 |  |  | 0.003 |

*(continued over)*

**Supplementary Table 6: Moderation Analysis for *AQP4* SNPs on Sleep Latency (*cont)*.**

|  |  |  | **Dominant**^†^ | |  |  |  |  | **Recessive**^†^ | |  |  |
| --- | --- | --- | --- | --- | --- | --- | --- | --- | --- | --- | --- | --- |
|  | **β** | **SE** | **Sig.** | **R^2^** | **Sig.** | **ΔR^2^** | **β** | **SE** | **Sig.** | **R^2^** | **Sig.** | **ΔR^2^** |
| *Model summary: rs491148* | | |  | 0.185 | <0.001 |  |  |  |  |  | 0.193 |  |
| Age | 0.010 | 0.004 | 0.016 |  |  |  |  | 0.012 | 0.004 | 0.005 |  |  |
| BMI | 0.005 | 0.006 | 0.360 |  |  |  |  | 0.005 | 0.006 | 0.393 |  |  |
| CVD risk | -0.018 | 0.039 | 0.650 |  |  |  |  | -0.017 | 0.039 | 0.657 |  |  |
| GDS | -0.011 | 0.015 | 0.450 |  |  |  |  | -0.011 | 0.015 | 0.459 |  |  |
| *APOE* ε4 | 0.316 | 0.057 | <0.001 |  |  |  |  | 0.320 | 0.057 | <0.001 |  |  |
| rs491148 | -0.035 | 0.075 | 0.639 |  |  |  |  | -0.333 | 0.271 | 0.220 |  |  |
| Latency | 0.002 | 0.002 | 0.639 |  |  |  |  | 0.004 | 0.001 | 0.014 |  |  |
| INT | **0.007** | **0.003** | **0.036** |  |  | **0.017** |  | **0.035** | **0.015** | **0.022** |  |  |

Model summary statistics for all Aquaporin 4 (*AQP4*) reference single nucleotide polymorphism (SNP) markers (rs). ^†^Genetic models: Dominant (heterozygote/homozygote for the minor allele (Mm or MM) vs homozygote for the major allele (mm)), Recessive (homozygote for the minor allele (MM) vs heterozygote/homozygote for the major allele (Mm/mm)); β, Coefficient of predictors: SE, standard error; Sig, p-value; R^2^, coefficient of multiple determination, ΔR^2^, multiple correlation coefficient (R) squared change; BMI, Body Mass Index; CVD risk, cardiovascular disease risk; GDS, Geriatric Depression Scale; *APOE*, Apolipoprotein E ε4 allele carriage (presence/absence); INT, Interaction (Sleep latency * model summary SNP). Models where the interaction term (INT) resulted in a statistically significant R^2^-change (p < 0.05) are indicated (bolded). If a model could not run due to that SNP variable being a constant then not applicable (N/A) is recorded for that model.

**Supplementary Table 7: Moderation Analysis for *AQP4* SNPs on Sleep Duration**

|  |  |  | **Dominant**^†^ | |  |  |  |  | **Recessive**^†^ | |  |  |
| --- | --- | --- | --- | --- | --- | --- | --- | --- | --- | --- | --- | --- |
|  | **β** | **SE** | **Sig.** | **R^2^** | **Sig.** | **ΔR^2^** | **β** | **SE** | **Sig.** | **R^2^** | **Sig.** | **ΔR^2^** |
| *Model summary: rs11661081* | | |  | 0.140 | <0.001 |  |  |  |  | 0.134 | <0.001 |  |
| Age | 0.011 | 0.004 | 0.010 |  |  |  | 0.011 | 0.004 | 0.012 |  |  |  |
| BMI | 0.004 | 0.006 | 0.500 |  |  |  | 0.004 | 0.006 | 0.477 |  |  |  |
| CVD risk | -0.009 | 0.040 | 0.823 |  |  |  | -0.007 | 0.041 | 0.856 |  |  |  |
| GDS | -0.010 | 0.015 | 0.537 |  |  |  | -0.007 | 0.015 | 0.640 |  |  |  |
| APOE ε4 | 0.304 | 0.058 | <0.001 |  |  |  | 0.301 | 0.059 | <0.001 |  |  |  |
| rs11661081 | -0.526 | 0.405 | 0.196 |  |  |  | -0.688 | 1.825 | 0.707 |  |  |  |
| Duration | -0.006 | 0.022 | 0.801 |  |  |  | 0.005 | 0.020 | 0.806 |  |  |  |
| INT | 0.074 | 0.057 | 0.199 |  |  | 0.007 | 0.083 | 0.258 | 0.748 |  |  | <0.001 |
| *Model summary rs9951307* | | |  | 0.143 | <0.001 |  |  |  |  | 0.139 | <0.001 |  |
| Age | 0.011 | 0.004 | 0.009 |  |  |  | 0.011 | 0.004 | 0.009 |  |  |  |
| BMI | 0.004 | 0.006 | 0.484 |  |  |  | 0.005 | 0.006 | 0.402 |  |  |  |
| CVD risk | -0.011 | 0.040 | 0.782 |  |  |  | -0.010 | 0.040 | 0.797 |  |  |  |
| GDS | -0.005 | 0.015 | 0.725 |  |  |  | -0.008 | 0.015 | 0.588 |  |  |  |
| APOE ε4 | 0.303 | 0.058 | <0.001 |  |  |  | 0.303 | 0.058 | <0.001 |  |  |  |
| rs9951307 | -0.174 | 0.299 | 0.561 |  |  |  | 0.301 | 0.416 | 0.470 |  |  |  |
| Duration | -0.003 | 0.034 | 0.920 |  |  |  | 0.011 | 0.022 | 0.622 |  |  |  |
| INT | 0.014 | 0.042 | 0.744 |  |  | <0.001 | -0.055 | 0.061 | 0.372 |  |  | 0.003 |
| *Model summary: rs7240333* | | |  | 0.151 | <0.001 |  |  |  |  | 0.147 | <0.001 |  |
| Age | 0.010 | 0.004 | 0.019 |  |  |  | 0.011 | 0.004 | 0.009 |  |  |  |
| BMI | 0.004 | 0.006 | 0.505 |  |  |  | 0.004 | 0.006 | 0.548 |  |  |  |
| CVD risk | -0.002 | 0.040 | 0.951 |  |  |  | -0.003 | 0.040 | 0.942 |  |  |  |
| GDS | -0.008 | 0.015 | 0.620 |  |  |  | -0.009 | 0.015 | 0.545 |  |  |  |
| APOE ε4 | 0.322 | 0.059 | <0.001 |  |  |  | 0.321 | 0.059 | <0.001 |  |  |  |
| rs7240333 | 0.321 | 0.372 | 0.390 |  |  |  | -0.462 | 1.035 | 0.656 |  |  |  |
| Duration | 0.010 | 0.023 | 0.665 |  |  |  | 0.002 | 0.021 | 0.938 |  |  |  |
| INT | -0.039 | 0.052 | 0.458 |  |  | 0.002 | 0.065 | 0.148 | 0.659 |  |  | 0.001 |

*(continued over)*

**Supplementary Table 7: Moderation Analysis for *AQP4* SNPs on Sleep Duration (*cont)*.**

|  |  |  | **Dominant**^†^ | |  |  |  |  | **Recessive**^†^ | |  |  |
| --- | --- | --- | --- | --- | --- | --- | --- | --- | --- | --- | --- | --- |
|  | **β** | **SE** | **Sig.** | **R^2^** | **Sig.** | **ΔR^2^** | **β** | **SE** | **Sig.** | **R^2^** | **Sig.** | **ΔR^2^** |
| *Model summary: rs68006382* | | |  | 0.138 | <0.001 |  |  |  |  | 0.142 | <0.001 |  |
| Age | 0.011 | 0.004 | 0.010 |  |  |  | 0.011 | 0.004 | 0.014 |  |  |  |
| BMI | 0.006 | 0.006 | 0.350 |  |  |  | 0.006 | 0.006 | 0.368 |  |  |  |
| CVD risk | -0.005 | 0.040 | 0.894 |  |  |  | -0.007 | 0.040 | 0.871 |  |  |  |
| GDS | -0.006 | 0.016 | 0.723 |  |  |  | -0.007 | 0.015 | 0.670 |  |  |  |
| APOE ε4 | 0.302 | 0.059 | <0.001 |  |  |  | 0.307 | 0.059 | <0.001 |  |  |  |
| rs68006382 | -0.021 | 0.348 | 0.951 |  |  |  | -0.621 | 0.776 | 0.425 |  |  |  |
| Duration | 0.002 | 0.024 | 0.923 |  |  |  | -0.001 | 0.021 | 0.958 |  |  |  |
| INT | 0.009 | 0.049 | 0.859 |  |  | <0.001 | 0.099 | 0.103 | 0.340 |  |  | 0.004 |
| *Model summary: rs71353406* | | |  | 0.135 | <0.001 |  |  |  |  | 0.144 | <0.001 |  |
| Age | 0.190 | 0.004 | 0.017 |  |  |  | 0.010 | 0.004 | 0.016 |  |  |  |
| BMI | 0.005 | 0.006 | 0.426 |  |  |  | 0.005 | 0.006 | 0.397 |  |  |  |
| CVD risk | -0.009 | 0.041 | 0.823 |  |  |  | -0.012 | 0.040 | 0.776 |  |  |  |
| GDS | -0.004 | 0.016 | 0.793 |  |  |  | -0.007 | 0.015 | 0.656 |  |  |  |
| APOE ε4 | 0.297 | 0.060 | <0.001 |  |  |  | 0.307 | 0.059 | <0.001 |  |  |  |
| rs71353406 | 0.190 | 0.305 | 0.535 |  |  |  | -0.679 | 0.578 | 0.241 |  |  |  |
| Duration | 0.013 | 0.028 | 0.634 |  |  |  | -0.005 | 0.021 | 0.830 |  |  |  |
| INT | -0.021 | 0.043 | 0.623 |  |  | 0.001 | 0.112 | 0.080 | 0.162 |  |  | 0.008 |
| *Model summary: rs12968026* | | |  | 0.149 | <0.001 |  |  |  |  | 0.126 | <0.001 |  |
| Age | 0.012 | 0.004 | 0.005 |  |  |  | 0.011 | 0.004 | 0.010 |  |  |  |
| BMI | 0.005 | 0.006 | 0.370 |  |  |  | 0.004 | 0.006 | 0.520 |  |  |  |
| CVD risk | -0.023 | 0.040 | 0.565 |  |  |  | -0.009 | 0.040 | 0.816 |  |  |  |
| GDS | -0.007 | 0.015 | 0.662 |  |  |  | -0.003 | 0.015 | 0.838 |  |  |  |
| *APOE* ε4 | 0.289 | 0.058 | <0.001 |  |  |  | 0.283 | 0.059 | <0.001 |  |  |  |
| rs12968026 | 0.807 | 0.352 | 0.023 |  |  |  | 0.065 | 0.715 | 0.928 |  |  |  |
| Duration | 0.026 | 0.023 | 0.251 |  |  |  | 0.005 | 0.021 | 0.817 |  |  |  |
| INT | **-0.104** | **0.049** | **0.034** |  |  | **0.019** | -0.010 | 0.105 | 0.923 |  |  | <0.001 |

*(continued over)*

**Supplementary Table 7: Moderation Analysis for *AQP4* SNPs on Sleep Duration (*cont)*.**

|  |  |  | **Dominant**^†^ | |  |  |  |  | **Recessive**^†^ | |  |  |
| --- | --- | --- | --- | --- | --- | --- | --- | --- | --- | --- | --- | --- |
|  | **β** | **SE** | **Sig.** | **R^2^** | **Sig.** | **ΔR^2^** | **β** | **SE** | **Sig.** | **R^2^** | **Sig.** | **ΔR^2^** |
| *Model summary: rs3875089* | | |  | 0.143 | <0.001 |  |  |  |  | 0.138 | <0.001 |  |
| Age | 0.011 | 0.004 | 0.008 |  |  |  | 0.011 | 0.004 | 0.008 |  |  |  |
| BMI | 0.004 | 0.006 | 0.464 |  |  |  | 0.004 | 0.006 | 0.477 |  |  |  |
| CVD risk | -0.016 | 0.400 | 0.694 |  |  |  | -0.014 | 0.040 | 0.737 |  |  |  |
| GDS | -0.008 | 0.015 | 0.588 |  |  |  | -0.008 | 0.015 | 0.603 |  |  |  |
| APOE ε4 | 0.297 | 0.058 | <0.001 |  |  |  | 0.309 | 0.059 | <0.001 |  |  |  |
| rs3875089 | 0.448 | 0.311 | 0.151 |  |  |  | -0.301 | 0.677 | 0.657 |  |  |  |
| Duration | 0.023 | 0.025 | 0.343 |  |  |  | 0.003 | 0.021 | 0.876 |  |  |  |
| INT | -0.056 | 0.044 | 0.200 |  |  | 0.007 | 0.064 | 0.098 | 0.515 |  |  | 0.002 |
| *Model summary: rs162007* | | |  | 0.133 | <0.001 |  |  |  |  | 0.134 | <0.001 |  |
| Age | 0.011 | 0.004 | 0.012 |  |  |  | 0.011 | 0.004 | 0.014 |  |  |  |
| BMI | 0.004 | 0.006 | 0.470 |  |  |  | 0.004 | 0.006 | 0.482 |  |  |  |
| CVD risk | -0.012 | 0.040 | 0.767 |  |  |  | -0.012 | 0.040 | 0.767 |  |  |  |
| GDS | -0.007 | 0.015 | 0.652 |  |  |  | -0.007 | 0.015 | 0.668 |  |  |  |
| APOE ε4 | 0.302 | 0.059 | <0.001 |  |  |  | 0.302 | 0.059 | <0.001 |  |  |  |
| rs162007 | -0.051 | 0.309 | 0.870 |  |  |  | -0.217 | 1.742 | 0.901 |  |  |  |
| Duration | 0.003 | 0.025 | 0.900 |  |  |  | 0.005 | 0.020 | 0.820 |  |  |  |
| INT | 0.007 | 0.043 | 0.872 |  |  | <0.001 | 0.022 | 0.275 | 0.936 |  |  | <0.001 |
| *Model summary: rs162003* | | |  | 0.144 | <0.001 |  |  |  |  | N/A |  |  |
| Age | 0.011 | 0.004 | 0.011 |  |  |  |  |  |  |  |  |  |
| BMI | 0.004 | 0.006 | 0.554 |  |  |  |  |  |  |  |  |  |
| CVD risk | -0.007 | 0.040 | 0.866 |  |  |  |  |  |  |  |  |  |
| GDS | -0.007 | 0.015 | 0.620 |  |  |  |  |  |  |  |  |  |
| APOE ε4 | 0.301 | 0.058 | <0.001 |  |  |  |  |  |  |  |  |  |
| rs162003 | 0.662 | 0.571 | 0.248 |  |  |  |  |  |  |  |  |  |
| Duration | 0.015 | 0.021 | 0.466 |  |  |  |  |  |  |  |  |  |
| INT | -0.100 | 0.077 | 0.194 |  |  | 0.007 |  |  |  |  |  |  |

*(continued over)*

**Supplementary Table 7: Moderation Analysis for *AQP4* SNPs on Sleep Duration (*cont)*.**

|  |  |  | **Dominant**^†^ | |  |  |  |  | **Recessive**^†^ | |  |  |
| --- | --- | --- | --- | --- | --- | --- | --- | --- | --- | --- | --- | --- |
|  | **β** | **SE** | **Sig.** | **R^2^** | **Sig.** | **ΔR^2^** | **β** | **SE** | **Sig.** | **R^2^** | **Sig.** | **ΔR^2^** |
| *Model summary: rs151245* | | |  | 0.142 | <0.001 |  |  |  |  | 0.137 | <0.001 |  |
| Age | 0.011 | 0.004 | 0.008 |  |  |  | 0.011 | 0.004 | 0.011 |  |  |  |
| BMI | 0.004 | 0.006 | 0.505 |  |  |  | 0.004 | 0.006 | 0.518 |  |  |  |
| CVD risk | -0.012 | 0.040 | 0.772 |  |  |  | -0.010 | 0.040 | 0.808 |  |  |  |
| GDS | -0.010 | 0.015 | 0.529 |  |  |  | -0.008 | 0.015 | 0.599 |  |  |  |
| APOE ε4 | 0.309 | 0.058 | <0.001 |  |  |  | 0.308 | 0.057 | <0.001 |  |  |  |
| rs151245 | 0.437 | 0.298 | 0.144 |  |  |  | -0.159 | 0.366 | 0.665 |  |  |  |
| Duration | 0.043 | 0.033 | 0.184 |  |  |  | -0.001 | 0.023 | 0.995 |  |  |  |
| INT | -0.062 | 0.042 | 0.140 |  |  | 0.009 | 0.031 | 0.052 | 0.547 |  |  | 0.002 |
| *Model summary: rs151246* | | |  | 0.135 | <0.001 |  |  |  |  | 0.134 | <0.001 |  |
| Age | 0.011 | 0.004 | 0.012 |  |  |  | 0.011 | 0.004 | 0.010 |  |  |  |
| BMI | 0.005 | 0.006 | 0.449 |  |  |  | 0.004 | 0.006 | 0.461 |  |  |  |
| CVD risk | -0.013 | 0.040 | 0.749 |  |  |  | -0.012 | 0.040 | 0.766 |  |  |  |
| GDS | -0.007 | 0.015 | 0.645 |  |  |  | -0.006 | 0.015 | 0.677 |  |  |  |
| APOE ε4 | 0.302 | 0.059 | <0.001 |  |  |  | 0.303 | 0.059 | <0.001 |  |  |  |
| rs151246 | -0.208 | 0.308 | 0.500 |  |  |  | 0.050 | 0.626 | 0.937 |  |  |  |
| Duration | -0.003 | 0.025 | 0.905 |  |  |  | 0.008 | 0.021 | 0.710 |  |  |  |
| INT | 0.026 | 0.044 | 0.559 |  |  | 0.001 | -0.016 | 0.082 | 0.843 |  |  | <0.001 |
| *Model summary: rs2339214* | | |  | 0.132 | <0.001 |  |  |  |  | 0.174 | <0.001 |  |
| Age | 0.010 | 0.004 | 0.018 |  |  |  | 0.011 | 0.004 | 0.009 |  |  |  |
| BMI | 0.005 | 0.006 | 0.403 |  |  |  | 0.004 | 0.006 | 0.507 |  |  |  |
| CVD risk | -0.011 | 0.041 | 0.796 |  |  |  | -0.009 | 0.040 | 0.819 |  |  |  |
| GDS | -0.005 | 0.016 | 0.774 |  |  |  | -0.008 | 0.015 | 0.595 |  |  |  |
| *APOE* ε4 | 0.302 | 0.059 | <0.001 |  |  |  | 0.307 | 0.058 | <0.001 |  |  |  |
| rs2339214 | 0.056 | 0.324 | 0.864 |  |  |  | -0.993 | 0.329 | 0.003 |  |  |  |
| Duration | 0.014 | 0.038 | 0.714 |  |  |  | -0.031 | 0.024 | 0.197 |  |  |  |
| INT | -0.009 | 0.045 | 0.850 |  |  | <0.001 | **0.149** | **0.047** | **0.002** |  |  | **0.041** |

*(continued over)*

**Supplementary Table 7: Moderation Analysis for *AQP4* SNPs on Sleep Duration (*cont)*.**

|  |  |  | **Dominant**^†^ | |  |  |  |  | **Recessive**^†^ | |  |  |
| --- | --- | --- | --- | --- | --- | --- | --- | --- | --- | --- | --- | --- |
|  | **β** | **SE** | **Sig.** | **R^2^** | **Sig.** | **ΔR^2^** | **β** | **SE** | **Sig.** | **R^2^** | **Sig.** | **ΔR^2^** |
| *Model summary: rs491148* | | |  | 0.156 | <0.001 |  |  |  |  | 0.146 | <0.001 |  |
| Age | 0.011 | 0.004 | 0.007 |  |  |  | 0.012 | 0.004 | 0.005 |  |  |  |
| BMI | 0.005 | 0.006 | 0.377 |  |  |  | 0.004 | 0.006 | 0.736 |  |  |  |
| CVD risk | -0.023 | 0.040 | 0.565 |  |  |  | -0.016 | 0.040 | 0.684 |  |  |  |
| GDS | -0.012 | 0.015 | 0.419 |  |  |  | -0.008 | 0.015 | 0.574 |  |  |  |
| *APOE* ε4 | 0.317 | 0.058 | <0.001 |  |  |  | 0.316 | 0.059 | <0.001 |  |  |  |
| rs491148 | 0.707 | 0.320 | 0.028 |  |  |  | -0.135 | 0.662 | 0.839 |  |  |  |
| Duration | 0.030 | 0.024 | 0.202 |  |  |  | 0.005 | 0.021 | 0.819 |  |  |  |
| INT | **-0.090** | **0.045** | **0.045** |  |  | **0.016** | 0.053 | 0.097 | 0.584 |  |  | 0.001 |

Model summary statistics for all Aquaporin 4 (*AQP4*) reference single nucleotide polymorphism (SNP) markers (rs). ^†^Genetic models: Dominant (heterozygote/homozygote for the minor allele (Mm or MM) vs homozygote for the major allele (mm)), Recessive (homozygote for the minor allele (MM) vs heterozygote/homozygote for the major allele (Mm/mm)); β, Coefficient of predictors: SE, standard error; Sig, p-value; R^2^, coefficient of multiple determination, ΔR^2^, multiple correlation coefficient (R) squared change; BMI, Body Mass Index; CVD risk, cardiovascular disease risk; GDS, Geriatric Depression Scale; *APOE*, Apolipoprotein E ε4 allele carriage (presence/absence); INT, Interaction (Sleep duration * model summary SNP). Models where the interaction term (INT) resulted in a statistically significant R^2^-change (p < 0.05) are indicated (bolded). If a model could not run due to that SNP variable being a constant then not applicable (N/A) is recorded for that model.

**Supplementary Table 8: Moderation Analysis for *AQP4* SNPs on Sleep Disturbances**

|  |  |  | **Dominant**^†^ | |  |  |  |  | **Recessive**^†^ | |  |  |
| --- | --- | --- | --- | --- | --- | --- | --- | --- | --- | --- | --- | --- |
|  | **β** | **SE** | **Sig.** | **R^2^** | **Sig.** | **ΔR^2^** | **β** | **SE** | **Sig.** | **R^2^** | **Sig.** | **ΔR^2^** |
| *Model summary: rs11661081* | | |  | 0.137 | <0.001 |  |  |  |  | 0.138 | <0.001 |  |
| Age | 0.011 | 0.004 | 0.013 |  |  |  | 0.010 | 0.004 | 0.014 |  |  |  |
| BMI | 0.004 | 0.006 | 0.553 |  |  |  | 0.004 | 0.006 | 0.533 |  |  |  |
| CVD risk | -0.004 | 0.040 | 0.931 |  |  |  | 0.001 | 0.041 | 0.987 |  |  |  |
| GDS | -0.005 | 0.015 | 0.766 |  |  |  | -0.005 | 0.015 | 0.732 |  |  |  |
| APOE ε4 | 0.304 | 0.058 | <0.001 |  |  |  | 0.302 | 0.058 | <0.001 |  |  |  |
| rs11661081 | -0.046 | 0.178 | 0.796 |  |  |  | -0.459 | 0.819 | 0.576 |  |  |  |
| Disturbances | -0.052 | 0.054 | 0.332 |  |  |  | -0.049 | 0.048 | 0.305 |  |  |  |
| INT | 0.027 | 0.116 | 0.814 |  |  | <0.001 | 0.233 | 0.515 | 0.652 |  |  | 0.001 |
| *Model summary rs9951307* | | |  | 0.154 | <0.001 |  |  |  |  | 0.140 | <0.001 |  |
| Age | 0.011 | 0.004 | 0.012 |  |  |  | 0.011 | 0.004 | 0.013 |  |  |  |
| BMI | 0.003 | 0.006 | 0.584 |  |  |  | 0.005 | 0.006 | 0.446 |  |  |  |
| CVD risk | -0.004 | 0.040 | 0.931 |  |  |  | -0.004 | 0.040 | 0.925 |  |  |  |
| GDS | -0.003 | 0.015 | 0.826 |  |  |  | -0.005 | 0.015 | 0.765 |  |  |  |
| APOE ε4 | 0.311 | 0.058 | <0.001 |  |  |  | 0.302 | 0.058 | <0.001 |  |  |  |
| rs9951307 | -0.276 | 0.143 | 0.055 |  |  |  | -0.215 | 0.253 | 0.395 |  |  |  |
| Disturbances | -0.140 | 0.081 | 0.085 |  |  |  | -0.053 | 0.050 | 0.287 |  |  |  |
| INT | 0.147 | 0.098 | 0.133 |  |  | 0.009 | 0.102 | 0.157 | 0.517 |  |  | 0.002 |
| *Model summary: rs7240333* | | |  | 0.151 | <0.001 |  |  |  |  | 0.151 | <0.001 |  |
| Age | 0.010 | 0.004 | 0.014 |  |  |  | 0.011 | 0.004 | 0.010 |  |  |  |
| BMI | 0.003 | 0.006 | 0.580 |  |  |  | 0.003 | 0.006 | 0.590 |  |  |  |
| CVD risk | 0.001 | 0.040 | 0.972 |  |  |  | 0.002 | 0.040 | 0.957 |  |  |  |
| GDS | -0.007 | 0.015 | 0.634 |  |  |  | -0.008 | 0.015 | <0.001 |  |  |  |
| APOE ε4 | 0.326 | 0.059 | <0.001 |  |  |  | 0.318 | 0.058 | <0.001 |  |  |  |
| rs7240333 | 0.056 | 0.175 | 0.749 |  |  |  | 0.458 | 0.628 | 0.467 |  |  |  |
| Disturbances | -0.036 | 0.053 | 0.500 |  |  |  | -0.033 | 0.048 | 0.491 |  |  |  |
| INT | -0.006 | 0.117 | 0.959 |  |  | <0.001 | -0.355 | 0.444 | 0.425 |  |  | 0.003 |

*(continued over)*

**Supplementary Table 8: Moderation Analysis for *AQP4* SNPs on Sleep Disturbances (*cont)*.**

|  |  |  | **Dominant**^†^ | |  |  |  |  | **Recessive**^†^ | |  |  |
| --- | --- | --- | --- | --- | --- | --- | --- | --- | --- | --- | --- | --- |
|  | **β** | **SE** | **Sig.** | **R^2^** | **Sig.** | **ΔR^2^** | **β** | **SE** | **Sig.** | **R^2^** | **Sig.** | **ΔR^2^** |
| *Model summary: rs68006382* | | |  | 0.145 | <0.001 |  |  |  |  | 0.142 | <0.001 |  |
| Age | 0.011 | 0.004 | 0.012 |  |  |  | 0.011 | 0.004 | 0.014 |  |  |  |
| BMI | 0.005 | 0.006 | 0.414 |  |  |  | 0.005 | 0.006 | 0.399 |  |  |  |
| CVD risk | 0.003 | 0.041 | 0.938 |  |  |  | -0.002 | 0.041 | 0.970 |  |  |  |
| GDS | -0.004 | 0.016 | 0.800 |  |  |  | -0.006 | 0.016 | 0.712 |  |  |  |
| APOE ε4 | 0.304 | 0.059 | <0.001 |  |  |  | 0.307 | 0.059 | <0.001 |  |  |  |
| rs68006382 | 0.200 | 0.166 | 0.232 |  |  |  | -0.150 | 0.452 | 0.740 |  |  |  |
| Disturbances | -0.014 | 0.055 | 0.798 |  |  |  | -0.047 | 0.050 | 0.347 |  |  |  |
| INT | -0.127 | 0.119 | 0.289 |  |  | 0.005 | 0.190 | 0.319 | 0.552 |  |  | 0.002 |
| *Model summary: rs71353406* | | |  | 0.139 | <0.001 |  |  |  |  | 0.139 | <0.001 |  |
| Age | 0.011 | 0.004 | 0.013 |  |  |  | 0.010 | 0.004 | 0.016 |  |  |  |
| BMI | 0.004 | 0.006 | 0.476 |  |  |  | 0.005 | 0.006 | 0.427 |  |  |  |
| CVD risk | -0.006 | 0.041 | 0.892 |  |  |  | -0.006 | 0.041 | 0.892 |  |  |  |
| GDS | -0.004 | 0.016 | 0.775 |  |  |  | -0.006 | 0.012 | 0.718 |  |  |  |
| APOE ε4 | 0.303 | 0.059 | <0.001 |  |  |  | 0.305 | 0.059 | <0.001 |  |  |  |
| rs71353406 | 0.146 | 0.141 | 0.302 |  |  |  | 0.234 | 0.325 | 0.472 |  |  |  |
| Disturbances | -0.001 | 0.065 | 0.988 |  |  |  | -0.034 | 0.050 | 0.489 |  |  |  |
| INT | -0.079 | 0.096 | 0.410 |  |  | 0.003 | -0.090 | 0.229 | 0.489 |  |  | 0.001 |
| *Model summary: rs12968026* | | |  | 0.366 | <0.001 |  |  |  |  | 0.129 | <0.001 |  |
| Age | 0.011 | 0.004 | 0.008 |  |  |  | 0.110 | 0.004 | 0.010 |  |  |  |
| BMI | 0.004 | 0.006 | 0.497 |  |  |  | 0.003 | 0.006 | 0.582 |  |  |  |
| CVD risk | -0.006 | 0.041 | 0.878 |  |  |  | -0.004 | 0.041 | 0.918 |  |  |  |
| GDS | -0.003 | 0.015 | 0.838 |  |  |  | -0.003 | 0.015 | 0.870 |  |  |  |
| APOE ε4 | 0.280 | 0.059 | <0.001 |  |  |  | 0.285 | 0.059 | <0.001 |  |  |  |
| rs12968026 | -0.012 | 0.178 | 0.946 |  |  |  | -0.245 | 0.553 | 0.658 |  |  |  |
| Disturbances | -0.041 | 0.052 | 0.361 |  |  |  | -0.038 | 0.048 | 0.425 |  |  |  |
| INT | 0.057 | 0.116 | 0.621 |  |  | 0.001 | 0.154 | 0.333 | 0.644 |  |  | 0.001 |

*(continued over)*

**Supplementary Table 8: Moderation Analysis for *AQP4* SNPs on Sleep Disturbances (*cont)*.**

|  |  |  | **Dominant**^†^ | |  |  |  |  | **Recessive**^†^ | |  |  |
| --- | --- | --- | --- | --- | --- | --- | --- | --- | --- | --- | --- | --- |
|  | **β** | **SE** | **Sig.** | **R^2^** | **Sig.** | **ΔR^2^** | **β** | **SE** | **Sig.** | **R^2^** | **Sig.** | **ΔR^2^** |
| *Model summary: rs3875089* | | |  | 0.141 | <0.001 |  |  |  |  | 0.141 | <0.001 |  |
| Age | 0.010 | 0.004 | 0.014 |  |  |  | 0.011 | 0.004 | 0.011 |  |  |  |
| BMI | 0.004 | 0.006 | 0.538 |  |  |  | 0.004 | 0.006 | 0.519 |  |  |  |
| CVD risk | -0.001 | 0.040 | 0.988 |  |  |  | -0.006 | 0.041 | 0.879 |  |  |  |
| GDS | -0.004 | 0.015 | 0.771 |  |  |  | -0.006 | 0.015 | 0.704 |  |  |  |
| APOE ε4 | 0.303 | 0.058 | <0.001 |  |  |  | 0.310 | 0.058 | <0.001 |  |  |  |
| rs3875089 | 0.124 | 0.149 | 0.407 |  |  |  | 0.362 | 0.462 | 0.435 |  |  |  |
| Disturbances | -0.032 | 0.057 | 0.584 |  |  |  | -0.044 | 0.048 | 0.356 |  |  |  |
| INT | -0.049 | 0.099 | 0.622 |  |  | 0.001 | -0.149 | 0.284 | 0.602 |  |  | 0.001 |
| *Model summary: rs162007* | | |  | 0.134 | <0.001 |  |  |  |  | 0.135 | <0.001 |  |
| Age | 0.011 | 0.004 | 0.013 |  |  |  | 0.010 | 0.004 | 0.015 |  |  |  |
| BMI | 0.004 | 0.006 | 0.528 |  |  |  | 0.004 | 0.006 | 0.539 |  |  |  |
| CVD risk | -0.004 | 0.040 | 0.926 |  |  |  | -0.003 | 0.040 | 0.938 |  |  |  |
| GDS | -0.005 | 0.015 | 0.739 |  |  |  | -0.006 | 0.015 | 0.695 |  |  |  |
| APOE ε4 | 0.304 | 0.058 | <0.001 |  |  |  | 0.304 | 0.058 | <0.001 |  |  |  |
| rs162007 | 0.015 | 0.144 | 0.919 |  |  |  | -0.302 | 0.523 | 0.564 |  |  |  |
| Disturbances | -0.043 | 0.058 | 0.467 |  |  |  | -0.500 | 0.048 | 0.295 |  |  |  |
| INT | -0.012 | 0.097 | 0.905 |  |  | <0.001 | 0.177 | 0.413 | 0.668 |  |  | 0.001 |
| *Model summary: rs162003* | | |  | 0.138 | <0.001 |  |  |  |  | N/A |  |  |
| Age | 0.010 | 0.004 | 0.019 |  |  |  |  |  |  |  |  |  |
| BMI | 0.003 | 0.006 | 0.659 |  |  |  |  |  |  |  |  |  |
| CVD risk | -0.001 | 0.040 | 0.978 |  |  |  |  |  |  |  |  |  |
| GDS | -0.005 | 0.015 | 0.720 |  |  |  |  |  |  |  |  |  |
| APOE ε4 | 0.299 | 0.058 | <0.001 |  |  |  |  |  |  |  |  |  |
| rs162003 | -0.189 | 0.220 | 0.391 |  |  |  |  |  |  |  |  |  |
| Disturbances | -0.058 | 0.050 | 0.245 |  |  |  |  |  |  |  |  |  |
| INT | 0.083 | 0.155 | 0.593 |  |  | 0.001 |  |  |  |  |  |  |

*(continued over)*

**Supplementary Table 8: Moderation Analysis for *AQP4* SNPs on Sleep Disturbances (*cont)*.**

|  |  |  | **Dominant**^†^ | |  |  |  |  | **Recessive**^†^ | |  |  |
| --- | --- | --- | --- | --- | --- | --- | --- | --- | --- | --- | --- | --- |
|  | **β** | **SE** | **Sig.** | **R^2^** | **Sig.** | **ΔR^2^** | **β** | **SE** | **Sig.** | **R^2^** | **Sig.** | **ΔR^2^** |
| *Model summary: rs151245* | | |  | 0.134 | <0.001 |  |  |  |  | 0.150 | <0.001 |  |
| Age | 0.010 | 0.004 | 0.014 |  |  |  | 0.010 | 0.004 | 0.016 |  |  |  |
| BMI | 0.004 | 0.006 | 0.543 |  |  |  | 0.003 | 0.006 | 0.570 |  |  |  |
| CVD risk | -0.004 | 0.041 | 0.931 |  |  |  | 0.003 | 0.041 | 0.950 |  |  |  |
| GDS | -0.006 | 0.015 | 0.701 |  |  |  | -0.008 | 0.015 | 0.599 |  |  |  |
| APOE ε4 | 0.304 | 0.058 | <0.001 |  |  |  | 0.301 | 0.058 | <0.001 |  |  |  |
| rs151245 | 0.085 | 0.153 | 0.580 |  |  |  | 0.261 | 0.206 | 0.206 |  |  |  |
| Disturbances | -0.006 | 0.094 | 0.951 |  |  |  | -0.028 | 0.051 | 0.586 |  |  |  |
| INT | -0.056 | 0.108 | 0.602 |  |  | 0.001 | -0.144 | 0.138 | 0.586 |  |  | 0.004 |
| *Model summary: rs151246* | | |  | 0.148 | <0.001 |  |  |  |  | 0.141 | <0.001 |  |
| Age | 0.011 | 0.004 | 0.011 |  |  |  | 0.011 | 0.004 | 0.012 |  |  |  |
| BMI | 0.005 | 0.006 | 0.407 |  |  |  | 0.004 | 0.006 | 0.509 |  |  |  |
| CVD risk | -0.008 | 0.040 | 0.844 |  |  |  | -0.005 | 0.041 | 0.898 |  |  |  |
| GDS | -0.009 | 0.015 | 0.547 |  |  |  | -0.004 | 0.015 | 0.779 |  |  |  |
| APOE ε4 | 0.300 | 0.058 | <0.001 |  |  |  | 0.306 | 0.059 | <0.001 |  |  |  |
| rs151246 | 0.171 | 0.146 | 0.243 |  |  |  | -0.124 | 0.392 | 0.753 |  |  |  |
| Disturbances | 0.010 | 0.061 | 0.872 |  |  |  | -0.048 | 0.048 | 0.318 |  |  |  |
| INT | -0.143 | 0.098 | 0.144 |  |  | 0.009 | 0.039 | 0.256 | 0.880 |  |  | <0.001 |
| *Model summary: rs2339214* | | |  | 0.140 | <0.001 |  |  |  |  | 0.136 | <0.001 |  |
| Age | 0.010 | 0.004 | 0.020 |  |  |  | 0.010 | 0.004 | 0.021 |  |  |  |
| BMI | 0.005 | 0.006 | 0.472 |  |  |  | 0.004 | 0.006 | 0.472 |  |  |  |
| CVD risk | 0.001 | 0.041 | 0.992 |  |  |  | 0.001 | 0.041 | 0.984 |  |  |  |
| GDS | -0.005 | 0.016 | 0.749 |  |  |  | -0.003 | 0.016 | 0.843 |  |  |  |
| APOE ε4 | 0.301 | 0.059 | <0.001 |  |  |  | 0.300 | 0.059 | <0.001 |  |  |  |
| rs2339214 | -0.124 | 0.167 | 0.459 |  |  |  | 0.064 | 0.173 | 0.711 |  |  |  |
| Disturbances | -0.101 | 0.091 | 0.270 |  |  |  | -0.038 | 0.053 | 0.470 |  |  |  |
| INT | 0.076 | 0.107 | 0.478 |  |  | 0.478 | -0.020 | 0.123 | 0.869 |  |  | <0.001 |

*(continued over)*

**Supplementary Table 8: Moderation Analysis for *AQP4* SNPs on Sleep Disturbances (*cont)*.**

|  |  |  | **Dominant**^†^ | |  |  |  |  | **Recessive**^†^ | |  |  |
| --- | --- | --- | --- | --- | --- | --- | --- | --- | --- | --- | --- | --- |
|  | **β** | **SE** | **Sig.** | **R^2^** | **Sig.** | **ΔR^2^** | **β** | **SE** | **Sig.** | **R^2^** | **Sig.** | **ΔR^2^** |
| *Model summary: rs491148* | | |  | 0.145 | <0.001 |  |  |  |  | 0.151 | <0.001 |  |
| Age | 0.011 | 0.004 | 0.012 |  |  |  | 0.012 | 0.004 | 0.004 |  |  |  |
| BMI | 0.004 | 0.006 | 0.473 |  |  |  | 0.004 | 0.006 | 0.506 |  |  |  |
| CVD risk | -0.004 | 0.041 | 0.916 |  |  |  | -0.013 | 0.040 | 0.744 |  |  |  |
| GDS | -0.007 | 0.015 | 0.626 |  |  |  | -0.008 | 0.015 | 0.590 |  |  |  |
| APOE ε4 | 0.305 | 0.058 | <0.001 |  |  |  | 0.319 | 0.058 | 0.590 |  |  |  |
| rs491148 | 0.070 | 0.162 | 0.666 |  |  |  | -0.050 | 0.401 | 0.901 |  |  |  |
| Disturbances | -0.056 | 0.056 | 0.317 |  |  |  | -0.055 | 0.048 | 0.248 |  |  |  |
| INT | 0.006 | 0.104 | 0.955 |  |  | <0.001 | 0.179 | 0.249 | 0.473 |  |  | 0.002 |

Model summary statistics for all Aquaporin 4 (*AQP4*) reference single nucleotide polymorphism (SNP) markers (rs). ^†^Genetic models: Dominant (heterozygote/homozygote for the minor allele (Mm or MM) vs homozygote for the major allele (mm)), Recessive (homozygote for the minor allele (MM) vs heterozygote/homozygote for the major allele (Mm/mm)); β, Coefficient of predictors: SE, standard error; Sig, p-value; R^2^, coefficient of multiple determination, ΔR^2^, multiple correlation coefficient (R) squared change; BMI, Body Mass Index; CVD risk, cardiovascular disease risk; GDS, Geriatric Depression Scale; *APOE*, Apolipoprotein E ε4 allele carriage (presence/absence); INT, Interaction (Sleep disturbances * model summary SNP). Models where the interaction term (INT) resulted in a statistically significant R^2^-change (p < 0.05) are indicated (bolded). If a model could not run due to that SNP variable being a constant then not applicable (N/A) is recorded for that model.

**Supplementary Table 9: Moderation Analysis for *AQP4* SNPs on Sleep Efficiency**

|  |  |  | **Dominant**^†^ | |  |  |  |  | **Recessive**^†^ | |  |  |
| --- | --- | --- | --- | --- | --- | --- | --- | --- | --- | --- | --- | --- |
|  | **β** | **SE** | **Sig.** | **R^2^** | **Sig.** | **ΔR^2^** | **β** | **SE** | **Sig.** | **R^2^** | **Sig.** | **ΔR^2^** |
| *Model summary: rs11661081* | | |  | 0.135 | <0.001 |  |  |  |  | N/A | 0.135 |  |
| Age | 0.011 | 0.004 | 0.012 |  |  |  |  |  |  |  |  |  |
| BMI | 0.004 | 0.006 | 0.530 |  |  |  |  |  |  |  |  |  |
| CVD risk | -0.008 | 0.040 | 0.842 |  |  |  |  |  |  |  |  |  |
| GDS | -0.005 | 0.015 | 0.735 |  |  |  |  |  |  |  |  |  |
| APOE ε4 | 0.304 | 0.058 | <0.001 |  |  |  |  |  |  |  |  |  |
| rs11661081 | -0.019 | 0.077 | 0.807 |  |  |  |  |  |  |  |  |  |
| Efficiency | -0.031 | 0.037 | 0.409 |  |  |  |  |  |  |  |  |  |
| INT | 0.018 | 0.078 | 0.818 |  |  | <0.001 |  |  |  |  |  |  |
| *Model summary rs9951307* | | |  | 0.151 | <0.001 |  |  |  |  | 0.142 | <0.001 |  |
| Age | 0.011 | 0.004 | 0.012 |  |  |  | 0.011 | 0.004 | 0.011 |  |  |  |
| BMI | 0.005 | 0.006 | 0.451 |  |  |  | 0.004 | 0.006 | 0.486 |  |  |  |
| CVD risk | -0.010 | 0.040 | 0.798 |  |  |  | -0.007 | 0.040 | 0.861 |  |  |  |
| GDS | -0.005 | 0.015 | 0.750 |  |  |  | -0.005 | 0.015 | 0.746 |  |  |  |
| APOE ε4 | 0.310 | 0.058 | <0.001 |  |  |  | 0.303 | 0.058 | <0.001 |  |  |  |
| rs9951307 | -0.132 | 0.061 | 0.033 |  |  |  | -0.064 | 0.100 | 0.522 |  |  |  |
| Efficiency | -0.097 | 0.057 | 0.087 |  |  |  | -0.026 | 0.035 | 0.464 |  |  |  |
| INT | 0.112 | 0.069 | 0.107 |  |  | 0.010 | 0.002 | 0.094 | 0.984 |  |  | <0.001 |
| *Model summary: rs7240333* | | |  | 0.151 | <0.001 |  |  |  |  | 0.148 | <0.001 |  |
| Age | 0.011 | 0.004 | 0.013 |  |  |  | 0.011 | 0.004 | 0.012 |  |  |  |
| BMI | 0.003 | 0.006 | 0.568 |  |  |  | 0.003 | 0.006 | 0.570 |  |  |  |
| CVD risk | -0.001 | 0.040 | 0.995 |  |  |  | -0.001 | 0.040 | 0.985 |  |  |  |
| GDS | -0.007 | 0.015 | 0.632 |  |  |  | -0.007 | 0.015 | 0.655 |  |  |  |
| APOE ε4 | 0.325 | 0.059 | <0.001 |  |  |  | 0.319 | 0.059 | <0.001 |  |  |  |
| rs7240333 | 0.064 | 0.075 | 0.392 |  |  |  | 0.017 | 0.263 | 0.949 |  |  |  |
| Efficiency | -0.012 | 0.037 | 0.742 |  |  |  | -0.017 | 0.033 | 0.603 |  |  |  |
| INT | -0.030 | 0.083 | 0.719 |  |  | 0.001 | -0.101 | 0.450 | 0.823 |  |  | <0.001 |

*(continued over)*

**Supplementary Table 9: Moderation Analysis for *AQP4* SNPs on Sleep Efficiency (*cont)*.**

|  |  |  | **Dominant**^†^ | |  |  |  |  | **Recessive**^†^ | |  |  |
| --- | --- | --- | --- | --- | --- | --- | --- | --- | --- | --- | --- | --- |
|  | **β** | **SE** | **Sig.** | **R^2^** | **Sig.** | **ΔR^2^** | **β** | **SE** | **Sig.** | **R^2^** | **Sig.** | **ΔR^2^** |
| *Model summary: rs68006382* | | |  | 0.140 | <0.001 |  |  |  |  | 0.146 | <0.001 |  |
| Age | 0.011 | 0.004 | 0.012 |  |  |  | 0.010 | 0.004 | 0.017 |  |  |  |
| BMI | 0.005 | 0.006 | 0.400 |  |  |  | 0.005 | 0.006 | 0.460 |  |  |  |
| CVD risk | -0.004 | 0.042 | 0.931 |  |  |  | -0.007 | 0.041 | 0.860 |  |  |  |
| GDS | -0.004 | 0.016 | 0.777 |  |  |  | -0.004 | 0.016 | 0.789 |  |  |  |
| APOE ε4 | 0.303 | 0.059 | <0.001 |  |  |  | 0.306 | 0.059 | <0.001 |  |  |  |
| rs68006382 | 0.051 | 0.070 | 0.469 |  |  |  | 0.232 | 0.186 | 0.215 |  |  |  |
| Efficiency | -0.018 | 0.040 | 0.661 |  |  |  | -0.018 | 0.034 | 0.587 |  |  |  |
| INT | -0.023 | 0.079 | 0.776 |  |  | <0.001 | -0.386 | 0.319 | 0.228 |  |  | 0.006 |
| *Model summary: rs71353406* | | |  | 0.139 | <0.001 |  |  |  |  | 0.144 | <0.001 |  |
| Age | 0.011 | 0.004 | 0.014 |  |  |  | 0.010 | 0.004 | 0.023 |  |  |  |
| BMI | 0.005 | 0.006 | 0.432 |  |  |  | 0.004 | 0.006 | 0.471 |  |  |  |
| CVD risk | -0.010 | 0.041 | 0.806 |  |  |  | -0.011 | 0.041 | 0.796 |  |  |  |
| GDS | -0.004 | 0.016 | 0.823 |  |  |  | -0.003 | 0.016 | 0.873 |  |  |  |
| APOE ε4 | 0.306 | 0.059 | <0.001 |  |  |  | 0.301 | 0.059 | <0.001 |  |  |  |
| rs71353406 | 0.071 | 0.062 | 0.256 |  |  |  | 0.209 | 0.134 | 0.121 |  |  |  |
| Efficiency | -0.001 | 0.047 | 0.980 |  |  |  | -0.017 | 0.034 | 0.612 |  |  |  |
| INT | -0.052 | 0.067 | 0.442 |  |  | 0.003 | -0.289 | 0.229 | 0.208 |  |  | 0.007 |
| *Model summary: rs12968026* | | |  | 0.136 | <0.001 |  |  |  |  | 0.128 | <0.001 |  |
| Age | 0.011 | 0.004 | 0.008 |  |  |  | 0.011 | 0.004 | 0.011 |  |  |  |
| BMI | 0.004 | 0.006 | 0.487 |  |  |  | 0.003 | 0.006 | 0.600 |  |  |  |
| CVD risk | -0.007 | 0.040 | 0.866 |  |  |  | -0.006 | 0.040 | 0.888 |  |  |  |
| GDS | -0.004 | 0.015 | 0.808 |  |  |  | -0.002 | 0.015 | 0.915 |  |  |  |
| APOE ε4 | 0.281 | 0.058 | <0.001 |  |  |  | 0.283 | 0.059 | <0.001 |  |  |  |
| rs12968026 | 0.043 | 0.075 | 0.568 |  |  |  | -0.030 | 0.207 | 0.717 |  |  |  |
| Efficiency | -0.032 | 0.036 | 0.384 |  |  |  | -0.026 | 0.034 | 0.439 |  |  |  |
| INT | 0.047 | 0.084 | 0.577 |  |  | 0.001 | 0.038 | 0.204 | 0.852 |  |  | <0.001 |

*(continued over)*

**Supplementary Table 9: Moderation Analysis for *AQP4* SNPs on Sleep Efficiency (*cont)*.**

|  |  |  | **Dominant**^†^ | |  |  |  |  | **Recessive**^†^ | |  |  |
| --- | --- | --- | --- | --- | --- | --- | --- | --- | --- | --- | --- | --- |
|  | **β** | **SE** | **Sig.** | **R^2^** | **Sig.** | **ΔR^2^** | **β** | **SE** | **Sig.** | **R^2^** | **Sig.** | **ΔR^2^** |
| *Model summary: rs3875089* | | |  | 0.139 | <0.001 |  |  |  |  | 0.139 | <0.001 |  |
| Age | 0.011 | 0.004 | 0.012 |  |  |  | 0.011 | 0.004 | 0.009 |  |  |  |
| BMI | 0.004 | 0.006 | 0.516 |  |  |  | 0.004 | 0.006 | 0.510 |  |  |  |
| CVD risk | -0.005 | 0.040 | 0.894 |  |  |  | -0.011 | 0.040 | 0.786 |  |  |  |
| GDS | -0.005 | 0.015 | 0.730 |  |  |  | -0.007 | 0.015 | 0.667 |  |  |  |
| APOE ε4 | 0.300 | 0.058 | <0.001 |  |  |  | 0.310 | 0.058 | <0.001 |  |  |  |
| rs3875089 | 0.053 | 0.067 | 0.425 |  |  |  | 0.176 | 0.179 | 0.326 |  |  |  |
| Efficiency | -0.025 | 0.038 | 0.508 |  |  |  | -0.024 | 0.033 | 0.326 |  |  |  |
| INT | -0.002 | 0.074 | 0.977 |  |  | <0.001 | -0.090 | 0.191 | 0.638 |  |  | 0.001 |
| *Model summary: rs162007* | | |  | 0.139 | <0.001 |  |  |  |  | 0.148 | <0.001 |  |
| Age | 0.011 | 0.004 | 0.012 |  |  |  | 0.010 | 0.004 | 0.016 |  |  |  |
| BMI | 0.003 | 0.006 | 0.568 |  |  |  | 0.004 | 0.006 | 0.480 |  |  |  |
| CVD risk | -0.008 | 0.040 | 0.848 |  |  |  | -0.005 | 0.040 | 0.893 |  |  |  |
| GDS | -0.004 | 0.015 | 0.811 |  |  |  | -0.008 | 0.015 | 0.620 |  |  |  |
| APOE ε4 | 0.301 | 0.058 | <0.001 |  |  |  | 0.305 | 0.058 | <0.001 |  |  |  |
| rs162007 | -0.036 | 0.061 | 0.555 |  |  |  | -0.508 | 0.308 | 0.100 |  |  |  |
| Efficiency | -0.049 | 0.041 | 0.229 |  |  |  | -0.033 | 0.033 | 0.323 |  |  |  |
| INT | 0.063 | 0.069 | 0.361 |  |  | 0.003 | 0.374 | 0.219 | 0.088 |  |  | 0.012 |
| *Model summary: rs162003* | | |  | 0.142 | <0.001 |  |  |  |  | N/A |  |  |
| Age | 0.010 | 0.004 | 0.015 |  |  |  |  |  |  |  |  |  |
| BMI | 0.003 | 0.006 | 0.660 |  |  |  |  |  |  |  |  |  |
| CVD risk | -0.010 | 0.040 | 0.808 |  |  |  |  |  |  |  |  |  |
| GDS | -0.005 | 0.015 | 0.751 |  |  |  |  |  |  |  |  |  |
| APOE ε4 | 0.301 | 0.058 | <0.001 |  |  |  |  |  |  |  |  |  |
| rs162003 | -0.111 | 0.084 | 0.186 |  |  |  |  |  |  |  |  |  |
| Efficiency | -0.039 | 0.034 | 0.257 |  |  |  |  |  |  |  |  |  |
| INT | 0.087 | 0.122 | 0.478 |  |  | 0.002 |  |  |  |  |  |  |

*(continued over)*

**Supplementary Table 9: Moderation Analysis for *AQP4* SNPs on Sleep Efficiency (*cont)*.**

|  |  |  | **Dominant**^†^ | |  |  |  |  | **Recessive**^†^ | |  |  |
| --- | --- | --- | --- | --- | --- | --- | --- | --- | --- | --- | --- | --- |
|  | **β** | **SE** | **Sig.** | **R^2^** | **Sig.** | **ΔR^2^** | **β** | **SE** | **Sig.** | **R^2^** | **Sig.** | **ΔR^2^** |
| *Model summary: rs151245* | | |  | 0.135 | <0.001 |  |  |  |  | 0.144 | <0.001 |  |
| Age | 0.010 | 0.004 | 0.014 |  |  |  | 0.011 | 0.004 | 0.009 |  |  |  |
| BMI | 0.004 | 0.006 | 0.538 |  |  |  | 0.003 | 0.006 | 0.646 |  |  |  |
| CVD risk | -0.007 | 0.040 | 0.856 |  |  |  | -0.005 | 0.040 | 0.896 |  |  |  |
| GDS | -0.006 | 0.015 | 0.714 |  |  |  | -0.007 | 0.015 | 0.662 |  |  |  |
| APOE ε4 | 0.304 | 0.058 | <0.001 |  |  |  | 0.305 | 0.058 | <0.001 |  |  |  |
| rs151245 | -0.001 | 0.063 | 0.997 |  |  |  | 0.123 | 0.087 | 0.159 |  |  |  |
| Efficiency | -0.035 | 0.059 | 0.554 |  |  |  | -0.012 | 0.035 | 0.738 |  |  |  |
| INT | 0.011 | 0.071 | 0.874 |  |  | <0.001 | -0.109 | 0.092 | 0.237 |  |  | 0.006 |
| *Model summary: rs151246* | | |  | 0.138 | <0.001 |  |  |  |  | 0.143 | <0.001 |  |
| Age | 0.011 | 0.004 | 0.010 |  |  |  | 0.011 | 0.004 | 0.009 |  |  |  |
| BMI | 0.004 | 0.006 | 0.507 |  |  |  | 0.003 | 0.006 | 0.615 |  |  |  |
| CVD risk | -0.008 | 0.040 | 0.839 |  |  |  | -0.007 | 0.040 | 0.868 |  |  |  |
| GDS | -0.006 | 0.015 | 0.677 |  |  |  | -0.006 | 0.015 | 0.689 |  |  |  |
| APOE ε4 | 0.297 | 0.059 | <0.001 |  |  |  | 0.305 | 0.058 | <0.001 |  |  |  |
| rs151246 | 0.002 | 0.064 | 0.971 |  |  |  | 0.066 | 0.161 | 0.680 |  |  |  |
| Efficiency | -0.004 | 0.044 | 0.927 |  |  |  | -0.018 | 0.034 | 0.599 |  |  |  |
| INT | -0.050 | 0.066 | 0.453 |  |  | 0.002 | -0.197 | 0.153 | 0.197 |  |  | 0.007 |
| *Model summary: rs2339214* | | |  | 0.137 | <0.001 |  |  |  |  | 0.139 | <0.001 |  |
| Age | 0.010 | 0.004 | 0.018 |  |  |  | 0.009 | 0.004 | 0.031 |  |  |  |
| BMI | 0.004 | 0.006 | 0.501 |  |  |  | 0.004 | 0.006 | 0.478 |  |  |  |
| CVD risk | -0.004 | 0.041 | 0.925 |  |  |  | -0.002 | 0.041 | 0.964 |  |  |  |
| GDS | -0.003 | 0.016 | 0.851 |  |  |  | -0.003 | 0.016 | 0.852 |  |  |  |
| APOE ε4 | 0.303 | 0.059 | <0.001 |  |  |  | 0.304 | 0.059 | <0.001 |  |  |  |
| rs2339214 | -0.024 | 0.070 | 0.735 |  |  |  | 0.068 | 0.072 | 0.348 |  |  |  |
| Efficiency | -0.062 | 0.067 | 0.355 |  |  |  | -0.024 | 0.039 | 0.546 |  |  |  |
| INT | 0.034 | 0.077 | 0.654 |  |  | 0.001 | -0.049 | 0.080 | 0.539 |  |  | 0.002 |

*(continued over)*

**Supplementary Table 9: Moderation Analysis for *AQP4* SNPs on Sleep Efficiency (*cont)*.**

|  |  |  | **Dominant**^†^ | |  |  |  |  | **Recessive**^†^ | |  |  |
| --- | --- | --- | --- | --- | --- | --- | --- | --- | --- | --- | --- | --- |
|  | **β** | **SE** | **Sig.** | **R^2^** | **Sig.** | **ΔR^2^** | **β** | **SE** | **Sig.** | **R^2^** | **Sig.** | **ΔR^2^** |
| *Model summary: rs491148* | | |  | 0.146 | <0.001 |  |  |  |  | 0.148 | <0.001 |  |
| Age | 0.011 | 0.004 | 0.012 |  |  |  | 0.012 | 0.004 | 0.005 |  |  |  |
| BMI | 0.005 | 0.006 | 0.454 |  |  |  | 0.004 | 0.006 | 0.515 |  |  |  |
| CVD risk | -0.009 | 0.040 | 0.815 |  |  |  | -0.014 | 0.040 | 0.729 |  |  |  |
| GDS | -0.011 | 0.016 | 0.483 |  |  |  | -0.007 | 0.015 | 0.626 |  |  |  |
| APOE ε4 | 0.305 | 0.058 | <0.001 |  |  |  | 0.317 | 0.058 | <0.001 |  |  |  |
| rs491148 | 0.030 | 0.068 | 0.654 |  |  |  | 0.231 | 0.174 | 0.186 |  |  |  |
| Efficiency | -0.046 | 0.038 | 0.224 |  |  |  | -0.027 | 0.033 | 0.416 |  |  |  |
| INT | 0.074 | 0.073 | 0.313 |  |  | 0.004 | -0.016 | 0.183 | 0.932 |  |  | <0.001 |

Model summary statistics for all Aquaporin 4 (*AQP4*) reference single nucleotide polymorphism (SNP) markers (rs). ^†^Genetic models: Dominant (heterozygote/homozygote for the minor allele (Mm or MM) vs homozygote for the major allele (mm)), Recessive (homozygote for the minor allele (MM) vs heterozygote/homozygote for the major allele (Mm/mm)); β, Coefficient of predictors: SE, standard error; Sig, p-value; R^2^, coefficient of multiple determination, ΔR^2^, multiple correlation coefficient (R) squared change; BMI, Body Mass Index; CVD risk, cardiovascular disease risk; GDS, Geriatric Depression Scale; *APOE*, Apolipoprotein E ε4 allele carriage (presence/absence); INT, Interaction (Sleep efficiency * model summary SNP). Models where the interaction term (INT) resulted in a statistically significant R^2^-change (p < 0.05) are indicated (bolded). If a model could not run due to that SNP variable being a constant then not applicable (N/A) is recorded for that model.
